# Cyclic Marinopyrrole Derivatives as Disruptors of Mcl-1 and Bcl-x_L_ Binding to Bim

**DOI:** 10.3390/md12031335

**Published:** 2014-03-07

**Authors:** Chunwei Cheng, Yan Liu, Maria E. Balasis, Nicholas L. Simmons, Jerry Li, Hao Song, Lili Pan, Yong Qin, K. C. Nicolaou, Said M. Sebti, Rongshi Li

**Affiliations:** 1 Key Laboratory of Drug Targeting and Drug Delivery Systems of the Ministry of Education and State Key Laboratory of Biotherapy, Department of Medicinal Natural Products, West China School of Pharmacy, Sichuan University, Chengdu 610041, China; E-Mails: chengchunwei666@163.com (C.C.); haoright@163.com (H.S.); pande179@163.com (L.P.); 2 Chemical Biology & Molecular Medicine Program, Department of Drug Discovery, H. Lee Moffitt Cancer Center and Research Institute, 12902 Magnolia Drive, Tampa, FL 33612, USA; E-Mails: maria.balasis@moffitt.org (M.E.B.); jerry.li@ucsf.edu (J.L.); said.sebti@moffitt.org (S.M.S.); 3 Department of Pharmaceutical Sciences, University of Nebraska Medical Center, 985965 Nebraska Medical Center, Omaha, NE 68198, USA; E-Mail: yan.liu@unmc.edu; 4 Department of Chemistry and The Skaggs Institute for Chemical Biology, The Scripps Research Institute, 10550 North Torrey Pines Road, La Jolla, CA 92037, USA; E-Mail: nsimmons@scripps.edu; 5 The Innovative Drug Research Centre, Chongqing University, Chongqing 400000, China; 6 Department of Chemistry, BioScience Research Collaborative, Rice University, 6500 Main Street, Houston, TX 77030, USA; E-Mail: kcn@rice.edu; 7 Department of Oncologic Sciences, Morsani College of Medicine, University of South Florida, 12901 Bruce B. Downs, Tampa, FL 33612, USA

**Keywords:** cyclic marinopyrroles, protein-protein interaction disruptors, apoptosis, SAR

## Abstract

A series of novel cyclic marinopyrroles were designed and synthesized. Their activity to disrupt the binding of the pro-apoptotic protein, Bim, to the pro-survival proteins, Mcl-1 and Bcl-x_L_, was evaluated using ELISA assays. Both atropisomers of marinopyrrole A (**1**) show similar potency. A tetrabromo congener **9** is two-fold more potent than **1**. Two novel cyclic marinopyrroles (**3** and **4**) are two- to seven-fold more potent than **1**.

## 1. Introduction

Marinopyrroles were first reported to show antibiotic activity against methicillin-resistant *Staphylococcus aureus* (MRSA) in 2008 by the Fenical group [1]. Due to their novel molecular structures and promising biological properties, marinopyrroles have attracted considerable attention [2,3,4,5,6,7,8,9,10,11]. We reported the first total synthesis of (±)-marinopyrrole A (**1**) along with 12 derivatives in early 2010 [3]. Synthesis of (±)-marinopyrrole A via an intermolecular Ullman coupling as the key step to form the bispyrrole system was published by Kanakis and Sarli five months later [4]. In 2011, the Nicolaou group published a new five-step method to access marinopyrrole derivatives, (+)-**1** and (–)-**1** atropisomers after a chiral separation of (±)-**1** using HPLC, as well as their antibiotic activities against MRSA [5]. In 2012, the Moore group published a biosynthetic approach toward marinopyrrole A via an *N*, *C*-bispyrrole homocoupling catalyzed by two flavin-dependent halogenases [8]. Last year, the total synthesis of marinopyrrole B and a review of the marinopyrroles were reported from the Clive group [9,10]. After we reported the synthesis of a novel series of “non-symmetrical” marinopyrrole derivatives and their antibiotic activities [6], we published optimization studies of the key step to avoid the formation of an oxazepine byproduct [7]. Most recently, we reported on the most potent marinopyrrole derivatives against MRSA [11]. Furthermore, we have also reported recently that (±)-marinopyrrole A antagonizes Mcl-1 and overcomes resistance of human cancer cells to the Bcl-x_L_ antagonist, ABT-737 [12]. During preparation of this manuscript, the discovery of a novel selective Mcl-1 small-molecule inhibitor blocking pancreatic cancer growth *in vitro* and *in vivo* resulted from high throughput screening followed by structure-based chemical optimization was reported recently by Nikolovska-Coleska and co-workers [13]. Here, we report on cyclic marinopyrroles as disruptors of protein-protein interactions between the pro-apoptotic protein, Bim, and the pro-survival proteins, Bcl-x_L_ and Mcl-1.

## 2. Results and Discussion

### 2.1. Design of Marinopyrrole Derivatives

**Figure 1:**
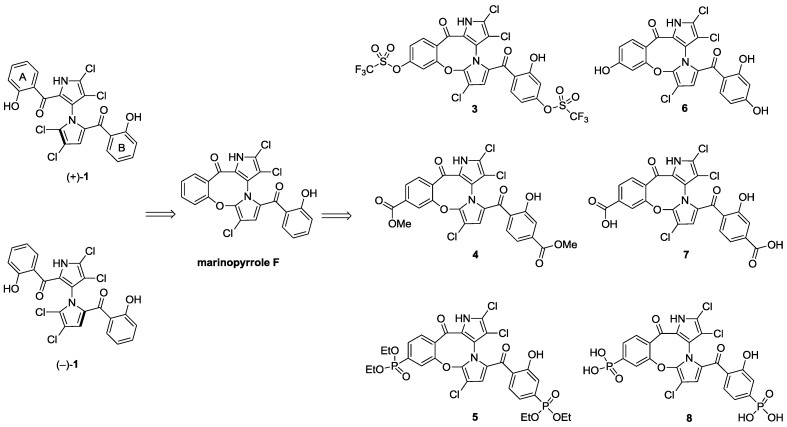
Structure of marinopyrrole A (**1**) and cyclic marinopyrroles **3**–**8**.

With the success of our synthetic studies on “symmetrical” marinopyrrole derivatives [3] bearing the same substitution on both A and B phenyl rings, we focused our attention on a series of non-symmetrical derivatives. In particular, marinopyrrole F (Figure 1) reported by Hughes *et al.* [2] was chosen as a starting point for optimization. Marinopyrrole F adopted specific conformations that locked one aromatic ring to the bispyrrole system, due to the fused eight-membered ether linkage, as shown by crystallographic X-ray analysis [2]. We were particularly interested in exploring the structure activity relationships (SARs) of the cyclic marinopyrroles with functional groups substituted on both aromatic rings, as shown in Figure 1. The introduction of substituents in the *para-*position relative to the carbonyl group on both aromatic rings, such as trifluoromethanesulfonate **3**, methyl ester **4** or diethyl phosphonate **5** functionality, would generate a series of compounds with the potential for a hydrogen bond (acceptor) and hydrophobic interactions with the target. Furthermore, the unmasked hydroxyl **6**, carboxylic acid **7** and phosphonic acid **8** groups in the corresponding positions could serve as both a hydrogen bond donor/acceptor and a functional group to improve aqueous solubility. To evaluate the potential differences in potency between the atropisomers of **1**, both (+)-**1** and (–)-**1** marinopyrrole A [5] were included in this study. The biological activity of brominated marinopyrrole A analog **9** [5] was also evaluated by ELISA assays.

### 2.2. Synthesis of Marinopyrrole Derivatives

**Scheme 1:**
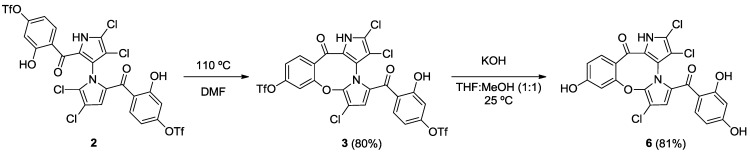
Synthesis of cyclic marinopyrroles **3** and **6**.

**Scheme 2:**
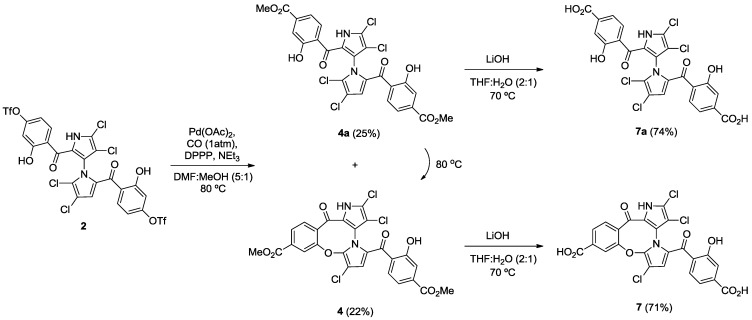
Synthesis of cyclic and symmetrical marinopyrroles.

**Scheme 3:**
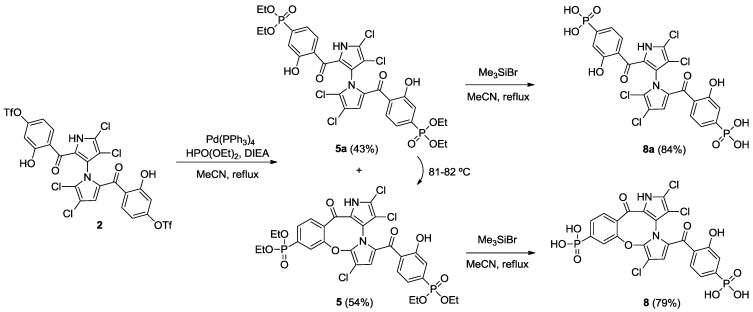
Synthesis of cyclic and symmetrical marinopyrroles.

Starting from our previously reported compound **2** [11], macrocycle **3** was obtained in an 80% yield after heating **2** in dimethylformamide (DMF) at 110 °C (Scheme 1). Removal of the trifluoromethanesulfonic groups by saponification in methanolic tetrahydrofuran (THF) gave phenol **6** in an 81% yield. Palladium-mediated carbonylation [14] of **2** provided symmetrical marinopyrrole **4a** and cyclic marinopyrrole **4** in a 25% and 22% yield, respectively. Further heating of compound **4a** at 80 °C generated **4**, presumably by spontaneous cyclization of 8-OH with 5′-Cl. Saponification of **4** and **4a** yielded the corresponding carboxylic acid derivatives, **7** and **7a**, respectively (Scheme 2). Palladium-catalyzed phosphorylation [15] of **2** with HPO(OEt)_2_ furnished a mixture of symmetrical marinopyrrole **5a** in a 43% yield, as well as cyclized **5** in a 54% yield (Scheme 3). Intramolecular cyclization of **5a** could also occur upon heating at 81–82 °C. Finally, upon treatment with Me_3_SiBr, **5** and **5a** could be smoothly converted to the corresponding bisphosphonic acids, **8** and **8a** [16]. 

### 2.3. Physicochemical Properties and SAR of the Marinopyrroles

**Figure 2:**
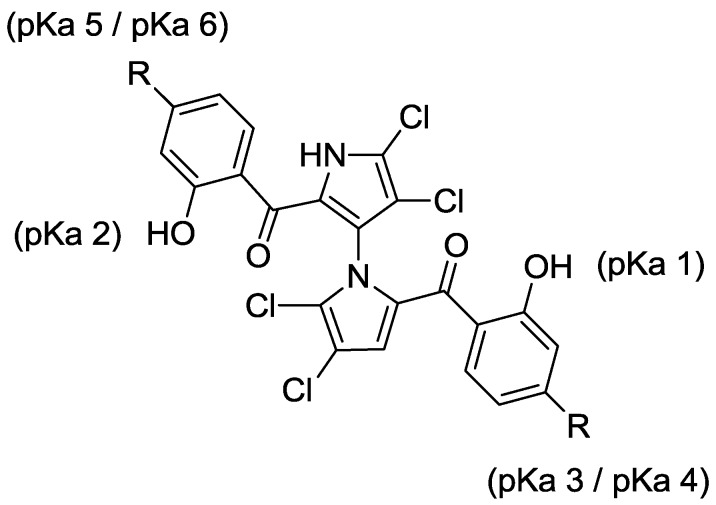
ELISA and physicochemical properties of **1** and symmetrical marinopyrroles.

Consistent with our previous report [12], the IC_50_ value of racemic marinopyrrole A to disrupt the binding of Mcl-1 to Bim was 8.9 μM. Although the activity of racemic marinopyrrole A against Bcl-x_L_/Bim binding was lower than we reported previously [12], this reflects the four times lower Bcl-x_L_ concentration utilized in our present assay. We observed no significant activity difference between atropisomers (+)-**1** and (–)-**1**, as both exhibited similar potencies against Mcl-1/Bim and Bcl-x_L_/Bim (Figure 2). Symmetrically *para*-substituted marinopyrroles with a carboxy methyl ester, **4a**, and diethyl phosphonate **5a** showed activity against Mcl-1/Bim, but were inactive against Bcl-x_L_/Bim (IC_50_ > 100 μM). Furthermore, substitution in the *para-*position of the carbonyl group with carboxylic acid **7a** showed lower activity than **1** against Mcl-1/Bim and little activity against Bcl-x_L_/Bim. Bisphosphonic acid marinopyrrole **8a** was slightly less potent than **1** against Mcl-1-Bim, but not Bcl-x_L_/Bim. Interestingly, the brominated marinopyrrole congener **9** [5] is two-fold more potent than **1** against both Mcl-1/Bim and Bcl-x_L_/Bim. 

Both p*K*_a_ and log *p* values were calculated using ChemAxon Software Version 5.12.3 [17,18]. The p*K*_a_ values of marinopyrrole A (**1**) are predicted to be 7.8 (p*K*_a_ 1) and 8.4 (p*K*_a_ 2), respectively (Figure 2). As reported previously [11], the difference in p*K*_a_ values for the hydroxyl group in ring A and ring B is presumably due to the axially chiral environment. The p*K*_a_ values of **1** are 1.6–2.2 log units lower than that of phenol (p*K*_a_ = 9.98 [19]). An equilibrium may exist between open conformations and closed conformations in **1**, similar to those observed in a recent report of intramolecular hydrogen bonding [20]. The Fenical group reported the X-ray structure of marinopyrrole B (3′-Br analogue of **1**) that indicated the preference for the formation of intramolecular hydrogen bonds between the *peri*-hydroxyl and the carbonyl group [1]. These intramolecular hydrogen bond interactions contribute to increasing not only compound acidity, but also its lipophilicity [20]. The calculated log *p* value of **1** is 5.6, which marginally violates the Rule of Five (RO5), drug-like properties formulated by Lipinski [21]. The calculated p*K*_a_ 1 and p*K*_a_ 2 values of marinopyrroles in Figure 2 range from 6.8 to 8.4. Compound **7a** has p*K*_a_ 3 (3.8) and p*K*_a_ 4 (3.2) values, due to the carboxylic acid, while **8a** has a p*K*_a_ 3 (0.7–5.5) and p*K*_a_ 4 (1.0–5.8) range of values corresponding to the phosphonic acid functional group. Clog *p* values of both compounds **7a** (4.6) and **8a** (2.4) reside within the suggested range for drug-like compounds. Despite the expected improvement in aqueous solubility of **7a** and **8a** over **1**, both were found to be less active against Mcl-1/Bim and Bcl-x_L_/Bim, perhaps due to unfavorable ionic and/or hydrogen bond interactions with the targets.

**Table:** 

Compound	Substituent	Mcl-1/Bim ^a^	Bcl-x_L_/Bim ^a^	p*K*_a_ 1 ^b^	p*K*_a_ 2 ^b^	p*K*_a_ 3/4 ^b^	p*K*_a_ 5/6 ^b^	Clog *p* ^b^
**(±)-1**	R = H	8.9 ± 1.0	16.4 ± 3.3	7.8	8.4	−	−	5.6
**(+)-1**	R = H	12.7 ± 1.0	19.7 ± 3.6	7.8	8.4	−	−	5.6
**(–)-1**	R = H	12.5 ± 1.4	12.0 ± 2.8	7.8	8.4	−	−	5.6
**4a**	R = COOMe	16.9 ± 2.3	>100	7.5	8.1	−	−	5.9
**5a**	R = PO(EtO)_2_	7.7 ± 2.2	>100	6.8	7.4	−	−	6.7
**7a**	R = COOH	61.4 ± 7.6	>100	7.8	8.4	3.8	3.2	4.6
**8a**	R = PO(OH)_2_	10.9 ± 3.1	27.3 ± 7.2	7.8	8.1	0.7/5.5 ^c^	1.0/5.8 ^c^	2.4
**9**	Tetrabromo-(±)-**1**	4.5 ± 0.9	7.3 ± 0.9	7.8	8.4	−	−	6.7

^a^ IC_50_ in micromolar (average ± SEM, *n* ≥ 3); ^b^ calculated using ChemAxon Software Version 5.12.3; ^c^ p*K*_a_ values from two hydroxyl groups.

**Figure 3:**
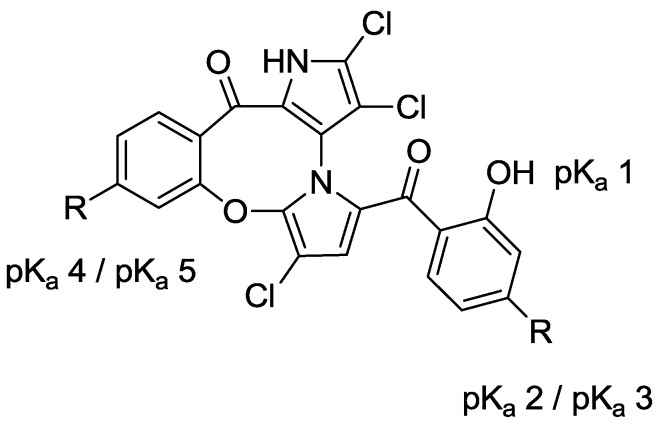
ELISA and physicochemical properties of cyclic marinopyrroles.

Compared to the SARs of the symmetrical marinopyrroles described (*vide supra*), cyclic marinopyrroles behaved similarly. Compounds containing functional groups with potential ionic and/or hydrogen bond interactions (**6**–**8**) reduce both anti-Mcl-1/Bim and Bcl-x_L_/Bim activity, as the cyclic marinopyrroles, phosphonic acid **8** and ester **5**, lack activity against both Mcl-1/Bim and Bcl-x_L_/Bim (IC_50_ > 100 μM in Figure 3). Conversely, methyl ester **4** is two-fold more potent than **1** against Mcl-1/Bim and seven-fold more potent against Bcl-x_L_/Bim. Interestingly, trifluoromethanesulfonate **3** is the most potent cyclic marinopyrrole, showing six- and seven-fold higher potency than **1** against Mcl-1/Bim and Bcl-x_L_/Bim, respectively. Compound **4** has a Clog *p* value of 4.7, while the most potent compound, **3**, has a Clog *p* value outside the advised range of RO5. The Clog *p* value for Compound **5** is marginally higher than the range of RO5, while the rest of the compounds (**6**–**8**) have Clog *p* values all within the recommended range for RO5. This series of cyclic marinopyrroles, which adopt constrained molecular geometries, due to the locked ring system [2], displayed an enhanced ability to disrupt the binding of Bim to Mcl-1 and Bcl-x_L_.

**Table:** 

Compound	Substituent	Mcl-1/Bim ^a^	Bcl-x_L_/Bim ^a^	p*K*_a_ 1 ^c^	p*K*_a_ 2/3 ^c^	p*K*_a_ 4/5 ^c^	Clog *p* ^c^
**3**	R = OSO_2_CF_3_	1.4 ± 0.3	2.3 ± 1.1	7.4	−	−	7.0
**4**	R = COOMe	4.3 ± 1.5	3.4 ± 0.9	7.8	−	−	4.7
**5**	R = PO(EtO)_2_	>100 ^b^	>100	7.1	−	−	5.5
**6**	R = OH	42.5 ± 6.0	>100	9.0	7.9	7.2	3.8
**7**	R = COOH	66.6 ± 2.6	>100	8.1	3.8	3.2	3.4
**8**	R = PO(OH)_2_	>100	>100	8.1	1.1/5.9 ^d^	0.6/5.5 ^d^	1.3

^a^ IC_50_ in micromolar (average ± SEM, *n* ≥ 3); ^b^ *n* = 2; ^c^ calculated using ChemAxon Software Version 5.12.3; ^d^ p*K*_a_ values from two hydroxyl groups.

**Figure 4:**
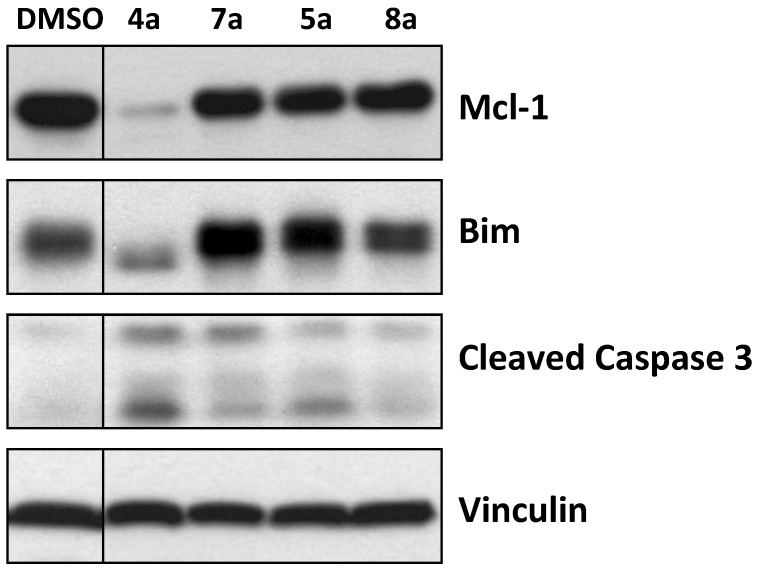
Effect of marinopyrroles on Mcl-1, Bim and caspase 3 in human breast cancer cells.

### 2.4. Activity in Intact Human Breast Cancer Cells

To determine if the marinopyrroles are active in intact cells, the human breast cancer MDA-MB-468 cells were treated with the marinopyrrole derivatives (10 μM for 16 h). The cells were then processed for Western blotting exactly as described by us previously [22]. Figure 4 shows that treatment of the cells with **4a** resulted in a significant decrease in the levels of Mcl-1 and Bim, and cleavage of caspase 3. Compound **7a**, the free carboxylic acid analogue of **4a**, did not decrease Mcl-1 and Bim and resulted in little caspase 3 cleavage. The phosphate **8a** and its corresponding ethyl ester **5a** had little effect on Mcl-1, Bim or caspase 3 (Figure 4). The (±)-marinopyrrole A (**1**) as reported by us previously [12] and its atropisomers, (+)-**1** and (–)-**1**, as well as **9**, tetrabromo-(±)-**1**, were able to decrease Mcl-1 and Bim and to cleave caspase 3 (data not shown). However, none of the cyclic marinopyrroles were active in intact cells (data not shown).

## 3. Experimental Section

### 3.1. Synthesis of Marinopyrrole Derivatives

All chemicals were purchased from commercial suppliers and used without further purification. All solvents were dried and distilled before use. Tetrahydrofuran was distilled from sodium/benzophenone. Dichloromethane and acetonitrile were distilled over calcium hydride. Flash column chromatography was performed with silica gel (200–300 mesh). ^1^H NMR spectra were recorded at either 400 MHz or 600 MHz at ambient temperature. ^13^C NMR spectra were recorded at either 100 or 150 MHz at ambient temperature. Infrared spectra were recorded on a Perkin-Elmer Spectrum 100 spectrometer. Copies of the NMR spectra of all the described compounds are provided in an Electronic Supporting Information (ESI) document. Melting points were determined with a melting point apparatus (Fukai X-4). High resolution mass spectra were performed by electrospray ionization (ESI) on an Agilent ESI-TOF LC-MS 6200 system. Analytical HPLC was performed on an Agilent 1100 series instrument with diode array detectors and auto samplers. All tested compounds possessed a purity of not less than 95%. ^1^H and ^13^C NMR spectra, and HPLC trace of the final compounds can be found in Supplementary Information (Figures S1–S30).

3-Hydroxy-4-(2,3,7-trichloro-13-oxo-10-(((trifluoromethyl)sulfonyl)oxy)-1,13 dihydrobenzo[*g*]di-pyrrolo[2,1-*b*:3′,2′-*d*][1,3]oxazocine-5-carbonyl)phenyl trifluoromethanesulfonate (**3**). Under N_2_, (4,4′,5,5′-tetrachloro-1′*H*-1,3′-bipyrrole-2,2′-diyl)bis(((2-hydroxy-4-hydroxytrifluoromethanesulfonate)-phenyl)methanone) (**2**) [11] (150 mg, 0.19 mmol) and NaI (120 mg, 0.75 mmol) were dissolved in DMF (5 mL). The mixture was heated to 110 °C and stirred for about 24 h. The reaction was quenched by the addition of saturated aqueous Na_2_S_2_O_3_ (20 mL) and extracted with EtOAc (ethyl acetate; 15 mL × 3). The suspension was filtered, and the filtrate was concentrated in vacuum. The residue was purified by flash column chromatography (16% EtOAc/petroleum ether, *R_f_* = 0.2) to give **3** (115 mg, 80%) as a light yellow solid. mp 94.8–96.4 °C; ^1^H NMR (400 MHz, CDCl_3_) δ 5.72 (s, 1H), 6.89 (dd, *J* = 8.8, 2.0 Hz, 1H), 6.98 (d, *J* = 2.4 Hz, 1H), 7.39 (dd, *J* = 8.8, 2.0 Hz, 1H), 7.73 (d, *J* = 2.4 Hz, 1H), 7.92 (d, *J* = 8.8 Hz, 1H), 8.14 (d, *J* = 8.8 Hz, 1H), 9.72 (*br* s, 1H), 11.55 (s, 1H) ppm; ^13^C NMR (CDCl_3_, 100 MHz) δ 101.68, 106.22, 111.40, 112.60, 116.88, 117.00, 119.02, 120.19, 120.98, 121.12, 121.28, 122.85, 124.18, 125.33, 129.73, 134.45, 135.18, 144.90, 152.81, 154.62, 157.16, 164.28, 174.98, 186.66 ppm; HRMS (M + H^+^) calcd. for C_24_H_10_Cl_3_F_6_N_2_O_10_S_2_ 768.8747, found 768.8809; IR (KBr): 3423, 3244, 2960, 2922, 2852, 1626, 1604, 1580, 1462, 1426, 1217, 1139, 1095, 965, 790 cm^−1^. HPLC purity, 99.1% (flow rate: 1 mL/min; column: Agilent ZORBAX 300SB-C8, 5 μm, 150 × 4.6 mm; wavelength: UV 254 nm; temperature: 25 °C; mobile phase: MeOH:H_2_O = 80:20; *t*_R_ = 28.9 min).

2,3,7-Trichloro-5-(2,4-dihydroxybenzoyl)-10-hydroxybenzo[*g*]dipyrrolo[2,1-*b*:3′,2′-*d*][1,3]oxazo-cin-13(1*H*)-one (**6**). To a solution of **3** (65 mg, 0.08 mmol) in a mixture of MeOH/THF (1:1, 4 mL) was added KOH (47 mg, 0.80 mmol) at room temperature. The mixture was heated to 70 °C and stirred for 10 h. The reaction mixture was adjusted to pH 7.0 with 0.5 N HCl and extracted with EtOAc (10 mL × 3). The combined organic layers were dried over anhydrous sodium sulfate, filtered and concentrated in vacuum. The residue was purified by flash column chromatography (33% EtOAc/petroleum ether, *R*_f_ = 0.3) to yield **6** (34 mg, 81%) as a yellow solid. mp 274.7–276.0 °C; ^1^H NMR (400 MHz, acetone-*d*_6_) δ 6.37 (s, 1H), 6.84 (d, *J* = 2.0 Hz, 1H), 6.95 (*br* s, 1H), 7.44 (dd, *J* = 8.8, 2.4 Hz, 1H), 7.72 (d, *J* = 2.0 Hz, 1H), 8.17 (dd, *J* = 8.4, 3.2 Hz, 1H), 8.38 (d, *J* = 8.4 Hz, 1H) ppm; ^13^C NMR (CD_3_OD, 100 MHz) δ 91.20, 94.31, 96.54, 100.56, 100.90, 105.20, 106.97, 109.25, 113.22, 113.86, 115.03, 115.39, 115.68, 125.60, 125.62, 127.17, 150.97, 156.11, 157.43, 158.23, 168.02 178.12 ppm; HRMS (M + K^+^) calcd. for C_22_H_11_Cl_3_KN_2_O_6_ 542.9320, found 542.9297; IR (KBr): 3415, 3251, 2962, 2924, 1619, 1581, 1547, 1476, 1456, 1310, 1090, 796 cm^−1^. HPLC purity, 98.9% (flow rate: 1 mL/min; column: Agilent ZORBAX 300SB-C8, 5 μm, 150 × 4.6 mm; wavelength: UV 254 nm; temperature: 25 °C; mobile phase: MeOH:H_2_O = 75:25; *t*_R_ = 7.5 min).

Methyl-2,3,7-trichloro-5-(2-hydroxy-4-(methoxycarbonyl)benzoyl)-13-oxo-1,13-dihydrobenzo[*g*]-dipyrrolo[2,1-*b*:3′,2′-*d*][1,3]oxazocine-10-carboxylate (**4**) and dimethyl-4,4′-(4,4′,5,5′-tetrachloro-1′*H*-[1,3′-bipyrrole]-2,2′-dicarbonyl)bis(3-hydroxybenzoate) (**4a**). Under CO (1 atm), **2** (400 mg, 0.50 mmol), DPPP (bis(diphenylphosphino)propane; 26 mg, 0.10 mmol), Pd(OAc)_2_ (11 mg, 0.05 mmol) and Et_3_N (251 mg, 2.50 mmol) were dissolved in a mixture of DMF/MeOH (5:1, 5 mL). The reaction was heated to 80 °C and stirred for 3 h. The reaction mixture was quenched with water (10 mL) and extracted with EtOAc (15 mL × 3). The combined organic layers were dried over anhydrous sodium sulfate, filtered and concentrated in vacuum. The residue was purified by flash column chromatography (50% EtOAc/petroleum ether, *R*_f_ = 0.2) to give **4** (70 mg, 22%) and **4a** (80 mg, 25%) as a pale yellow solid. 

**4**: mp 135.7–137.0 °C; ^1^H NMR (400 MHz, CDCl_3_) δ 3.96 (s, 3H), 3.98 (s, 3H), 5.72 (s, 1H), 7.56 (d, *J* = 8.0 Hz, 1H), 7.69 (s, 1H), 7.84 (d, *J* = 6.8 Hz, 1H), 8.07 (s, 2H), 8.40 (s, 1H), 9.88 (s, 1H), 11.22 (s, 1H) ppm; ^13^C NMR (acetone-*d*_6_, 100 MHz) δ 52.81, 53.16, 107.56, 118.58, 118.60, 120.76, 124.61, 128.87, 132.99, 132.99, 133.71, 134.02, 134.26, 136.82, 136.99, 149.00, 157.40, 157.51, 164.91, 165.33, 166.12, 167.90, 174.87, 176.18, 178.01, 183.00 ppm; HRMS (M + H^+^) calcd. for C_26_H_16_Cl_3_N_2_O_8_ 588.9972, found 588.9967; IR (KBr): 3416, 3236, 2954, 2852, 1730, 1609, 1580, 1461, 1414, 1288, 1207, 1090, 988, 806 cm^−1^. HPLC purity, 95.6% (flow rate: 1 mL/min; column: Waters C18, 5 μm, 150 × 4.6 mm; wavelength: UV 254 nm; temperature: 25 °C; mobile phase: MeOH:H_2_O = 90:10; *t*_R_ = 4.6 min).

**4a**: mp 99.4–101.0 °C; ^1^H NMR (400 MHz, acetone-*d_6_*) δ 3.84 (s, 3H), 3.86 (s, 3H), 6.18 (s, 1H), 7.27 (dd, *J* = 8.4, 1.6 Hz, 1H), 7.42–7.44 (m, 3H), 7.87 (d, *J* = 8.4 Hz, 1H), 8.04 (d, *J* = 8.0 Hz, 1H) ppm; ^13^C NMR (acetone-*d*_6_, 100 MHz) δ 52.68, 52.75, 110.16, 118.28, 118.51, 119.94, 120.60, 122.61, 123.20, 124.43, 126.43, 126.59, 127.10, 128.72, 130.72, 133.06, 135.43, 136.51, 158.90, 159.95, 166.17, 166.17, 184.78, 185.08, 185.60, 186.13 ppm; HRMS (M + H^+^) calcd. for C_26_H_17_Cl_4_N_2_O_8_ 624.9739, found 624.9736; IR (KBr): 3,245, 2,954, 1,727, 1,632, 1,599, 1,441, 1,291, 1,223, 1,093, 884, 759, 672 cm^−1^. HPLC purity, 96.3% (flow rate: 1 mL/min; column: Agilent ZORBAX 300SB-C8, 5 μm, 150 × 4.6 mm; wavelength: UV 254 nm; temperature: 25 °C; mobile phase: MeOH:H_2_O = 80:20; *t*_R_ = 6.6 min).

5-(4-Carboxy-2-hydroxybenzoyl)-2,3,7-trichloro-13-oxo-1,13-dihydrobenzo[*g*]dipyrrolo[2,1-*b*:3′,2′-*d*][1,3]oxazocine-10-carboxylic acid (**7**). To a solution of **4** (44 mg, 0.07 mmol) in a mixture of H_2_O/THF (1:2, 5 mL) was added LiOH (27 mg, 1.1 mmol) at room temperature. The reaction was heated to 70 °C and stirred for 10 h. The reaction mixture was adjusted to pH 5.0 with 0.5 N HCl and extracted with EtOAc (10 mL × 3). The combined organic layers were dried over anhydrous sodium sulfate, filtered and concentrated in vacuum. The residue was purified by reverse-phase flash column chromatography (6% AcOH, 23% H_2_O, 71% MeOH, *R*_f_ = 0.2) to give **7** (30 mg, 71%) as a light yellow solid. mp 215.5–217.0 °C; ^1^H NMR (400 MHz, DMSO-*d*_6_) δ 6.03 (s, 1H), 7.40 (m, 3H), 8.03 (m, 2H), 8.14 (s, 1H) ppm; ^13^C NMR (DMSO-*d*_6_, 100 MHz) δ 100.08, 107.72, 117.52, 120.03, 120.62, 123.74, 124.50, 124.73, 125.18, 128.83, 130.17, 130.78, 132.98, 133.43, 136.00, 139.29, 145.72, 156.81, 156.81, 166.57, 167.44, 173.04, 175.98, 183.02 ppm; HRMS (M + H^+^) calcd. for C_24_H_12_Cl_3_N_2_O_8_ 560.9659, found 560.9669; IR (KBr): 3420, 3240, 3127, 2925, 2600, 1710, 1604, 1580, 1462, 1413, 1311, 1210, 1025, 996, 906, 799, 761 cm^−1^. HPLC purity, 99.3% (flow rate: 1 mL/min; column: Waters C18, 5 μm, 150 × 4.6 mm; wavelength: UV 254 nm; temperature: 25 °C; mobile phase: MeOH:H_2_O = 55:45; *t*_R_ = 6.7 min).

4,4′-(4,4′,5,5′-Tetrachloro-1′*H*-[1,3′-bipyrrole]-2,2′-dicarbonyl)bis(3-hydroxybenzoic acid) (**7a**). To a solution of **4a** (27 mg, 0.04 mmol) in a mixture of H_2_O/THF (1:2, 3 mL) was added LiOH (16 mg, 0.65 mmol) at room temperature. The reaction was heated to 70 °C and stirred for 10 h. The reaction mixture was adjusted to pH 5.0 with 0.5 N HCl and extracted with EtOAc (10 mL × 3). The combined organic layers were dried over anhydrous sodium sulfate, filtered and concentrated in vacuum. The residue was purified by reverse-phase flash column chromatography (6% AcOH, 30% H_2_O, 64% MeOH, *R*_f_ = 0.2) to give **7a** (19 mg, 74%) as a light yellow solid. mp 190.5–192.0 °C; ^1^H NMR (400 MHz, DMSO-*d*_6_) δ 6.10 (s, 1H), 7.19 (d, *J* = 8.0 Hz, 2H), 7.29–7.33 (m, 4H) ppm; ^13^C NMR (DMSO-*d*_6_, 100 MHz) δ 109.69, 110.24, 116.80, 117.21, 118.60, 118.66, 120.03, 122.32, 122.57, 124.96, 129.10, 129.26, 129.80, 129.90, 129.92, 134.57, 135.43, 156.09, 156.47, 167.58, 167.58, 172.66, 181.88, 183.14 ppm; HRMS (M + Na^+^) calcd. for C_24_H_12_Cl_4_N_2_NaO_8_ 618.9245, found 618.9258; IR (KBr): 3075, 2956, 2919, 2851, 1707, 1631, 1599, 1446, 1394, 1294, 1228, 1023, 995, 885, 760 cm^−1^. HPLC purity, 98.6% (flow rate: 1 mL/min; column: Waters C18, 5 μm, 150 × 4.6 mm; wavelength: UV 254 nm; temperature: 25 °C; mobile phase: MeOH:H_2_O = 65:35; *t*_R_ = 5.1 min).

3-Hydroxy-4-(2,3,7-trichloro-13-oxo-10-(diethylphosphonyl)-1,13-dihydrobenzo[*g*]dipyrrolo[2,1-*b*:3′,2′-*d*][1,3]oxazocine-5-carbonyl)diethyl phosphonate (**5**) and tetraethyl((4,4′,5,5′-tetrachloro-1′*H*-[1,3′-bipyrrole]-2,2′-dicarbonyl)bis(3-hydroxy-4,1-phenylene))bis(phosphonate) (**5a**). Under N_2_, **2** (50 mg, 0.06 mmol), diethyl phosphonate (52 mg, 0.36 mmol), Pd(PPh_3_)_4_ (7.6 mg, 0.006 mmol) and *i*-Pr_2_NEt (48 mg, 0.36 mmol) were dissolved in anhydrous MeCN (5 mL). The reaction was heated to reflux and stirred for 10 h. The reaction mixture was quenched with water (10 mL) and extracted with EtOAc (15 mL × 3). The combined organic layers were dried over anhydrous sodium sulfate, filtered and concentrated in vacuum. The residue was purified by flash column chromatography (50% EtOAc/petroleum ether, *R*_f_ = 0.2) to give **5** (25 mg, 54%) and **5a** (20 mg, 43%) as a yellow solid. 

**5**: mp 122.8–124.3 °C; ^1^H NMR (400 MHz, CDCl_3_) δ 1.36 (t, *J* = 6.8 Hz, 12H), 4.12–4.24 (m, 8H), 5.71 (s, 1H), 7.36 (t, *J* = 8.4 Hz, 1H), 7.47 (d, *J* = 15.2 Hz, 1H), 7.87 (m, 2H), 8.08 (dd, *J* = 7.6, 5.2 Hz, 1H), 8.17 (d, *J* = 13.6 Hz, 1H), 9.85 (*br* s, 1H), 11.27 (s, 1H) ppm; ^13^C NMR (CDCl_3_, 100 MHz) δ 16.02, 16.02, 16.27, 16.27, 62.64, 62.70, 62.83, 62.83, 101.22, 106.12, 121.00, 121.59, 121.74, 123.06, 124.32, 125.10, 126.46, 130.33, 132.61, 132.77, 132.89, 135.07, 135.93, 136.94, 137.75, 145.48, 156.50, 161.50, 175.82, 187.12 ppm; HRMS (M + H^+^) calcd. for C_30_H_30_Cl_3_N_2_O_10_P_2_ 745.0441, found 745.0454; IR (KBr): 3421, 3338, 3123, 3078, 2983, 2925, 2855, 1614, 1579, 1461, 1258, 1232, 1050, 1021, 796 cm^−1^. HPLC purity, 97.2% (flow rate: 1 mL/min; column: Agilent ZORBAX 300SB-C8, 5 μm, 150 × 4.6 mm; wavelength: UV 254 nm; temperature: 25 °C; mobile phase: MeOH:H_2_O = 80:20; *t*_R_ = 6.8 min).

**5a**: mp 100.7–101.5 °C; ^1^H NMR (400 MHz, CDCl_3_) δ1.24–1.36 (m, 12H), 3.98–4.22 (m, 8H), 6.15 (s, 1H), 6.91 (dd, *J* = 11.6, 8.0 Hz, 1H), 7.24–7.27 (m, 1H), 7.30 (d, *J* = 14.4 Hz, 1H), 7.40 (d, *J* = 14.8 Hz, 1H), 7.52 (t, *J* = 14.4 Hz, 1H), 7.56 (dd, *J* = 7.6, 2.8 Hz, 1H), 8.00 (*br* s, 1H), 11.12 (s, 1H), 11.44 (*br* s, 1H) ppm; ^13^C NMR (CDCl_3_, 100 MHz) δ 16.22, 16.22, 16.28, 16.28, 62.69, 62.69, 62.74, 62.74, 108.90, 111.95, 117.68, 120.73, 121.03, 121.38, 121.47, 121.59, 122.07, 122.65, 122.84, 124.79, 130.95, 133.35, 135.84, 137.67, 160.29, 160.48, 161.32, 161.52, 185.51, 187.50 ppm; HRMS (M + H^+^) calcd. for C_30_H_31_Cl_4_N_2_O_10_P_2_ 781.0208, found 781.0220; IR (KBr): 3416, 3214, 2964, 2926, 2867, 1631, 1449, 1406, 1259, 1222, 1022, 938, 800, 671 cm^−1^. HPLC purity, 97.0% (flow rate, 1 mL/min; column: Phenomenex C6-phenyl, 5 μm, 150 × 4.6 mm; wavelength: UV 254 nm; temperature: 25 °C; mobile phase: MeOH:H_2_O = 80:20; *t*_R_= 4.0 min).

3-Hydroxy-4-(2,3,7-trichloro-13-oxo-10-phosphoryl-1,13-dihydrobenzo[*g*]dipyrrolo[2,1-*b*:3′,2′-*d*][1,3]oxazocine-5-carbonyl) phosphonic acid (**8**). To a solution of **5** (40 mg, 0.054 mmol) in MeCN (3 mL) was added Me_3_SiBr (230 mg, 1.50 mmol) via a syringe at room temperature under N_2_. The reaction was heated to reflux and stirred for 24 h. The reaction mixture was concentrated in vacuum. The residue was purified by reverse-phase flash column chromatography (6% AcOH, 47% H_2_O, 47% MeOH, *R*_f_ = 0.2) to give **8** (27 mg, 79%) as a yellow solid. mp 314.7–316.0 °C; ^1^H NMR (400 MHz, CD_3_OD) δ 5.95 (s, 1H), 7.26 (dd, *J* = 12.8, 8.4 Hz, 1H), 7.32 (d, *J* = 14.8 Hz, 1H), 7.53 (dd, *J* = 8.0, 4.4 Hz, 1H), 7.85 (dd, *J* = 12.8, 8.0 Hz, 1H), 8.08 (dd, *J* = 8.0, 4.4 Hz, 1H), 8.15 (d, *J* = 13.6 Hz, 1H) ppm; ^13^C NMR (CD_3_OD, 100 MHz) δ 101.57, 101.69, 106.80, 107.56, 120.56, 121.47, 122.50, 124.67, 125.16, 125.52, 125.87, 126.60, 127.10, 130.67, 132.50, 133.46, 136.20, 141.71, 147.04, 162.26, 177.27, 186.48 ppm; HRMS (M + H^+^) calcd. for C_22_H_14_Cl_3_N_2_O_10_P_2_ 632.9189, found 632.9193; IR (KBr): 3,790, 3,407, 2,955, 2,920, 2,850, 1,727, 1,596, 1,458, 1,401, 877 cm^−1^. HPLC purity, 99.7% (flow rate: 1 mL/min; column: Agilent ZORBAX 300SB-C8, 5 μm, 150 × 4.6 mm; wavelength: UV 254 nm; temperature: 25 °C; mobile phase: MeOH:H_2_O = 55:45; *t*_R_ = 4.1 min).

((4,4′,5,5′-Tetrachloro-1′*H*-[1,3′-bipyrrole]-2,2′-dicarbonyl)bis(3-hydroxy-4,1-phenylene))diphosphonic acid (**8a**). To a solution of **5a** (18 mg, 0.023 mmol) in MeCN (3 mL) was added Me_3_SiBr (99 mg, 0.65 mmol) via a syringe at room temperature under N_2_. The reaction was heated to reflux and stirred for 24 h. The reaction mixture was concentrated in vacuum. The residue was purified by reverse-phase flash column chromatography (6% AcOH, 30% H_2_O, 64% MeOH, *R*_f_ = 0.2) to give **8a** (13 mg, 84%) as a yellow solid. mp 317.6–318.7 °C; ^1^H NMR (400 MHz, CD_3_OD) δ 6.29 (s, 1H), 7.05 (s, 1H), 7.26–7.37 (m, 5H) ppm; ^13^C NMR (CD_3_OD, 100 MHz) δ 110.32, 110.38, 112.63, 114.07, 118.96, 120.50, 121.60, 122.41, 123.23, 123.83, 125.59, 125.94, 126.34, 127.43, 129.35, 130.79, 132.80, 136.61, 159.27, 159.82, 185.99, 187.14 ppm; HRMS (M + H^+^) calcd. for C_22_H_15_Cl_4_N_2_O_10_P_2_ 668.8956, found 668.8958; IR (KBr): 2,955, 2,919, 2,850, 1,626, 1,464, 1,020, 799 cm^−1^. HPLC purity, 99.5% (flow rate: 1 mL/min; column: Agilent ZORBAX 300SB-C8, 5 μm, 150 × 4.6 mm; wavelength: UV 254 nm; temperature: 25 °C; mobile phase: MeOH:H_2_O = 55:45; *t*_R_ = 4.0 min).

### 3.2. Enzyme-Linked Immunosorbent Assay (ELISA) and Western Blotting Following Treatment of Intact Human Breast Cancer Cells

ELISAs were performed using a similar procedure as previously described [12]. Briefly, 40 nM of biotinylated Bim BH3 peptide (Biomatik, Wilmington, DE, USA) in SuperBlock blocking buffer (Thermo Scientific Pierce, Rockford, IL, USA) was incubated in high-binding capacity streptavidin-coated plates (Thermo Scientific Pierce, Rockford, IL, USA) for 2 h. Compounds were diluted in 120 µL of PBS containing 10 nM of GST-Mcl-1 or GST-Bcl-x_L_ in 1.5-mL tubes for 15 min. Wells were washed with wash buffer (PBS containing 0.05% Tween-20) and then 100 µL of the compound/GST-protein mixture was transferred to the wells. The plates were incubated for 2 h, and then, the wells were washed with wash buffer. HRP-conjugated anti-GST antibody (Bethyl Laboratories, Montgomery, TX, USA) was diluted 1:2000 in SuperBlock, and 100 µL were transferred to each well. The plate was incubated for 1 h, and then, the wells were washed with wash buffer followed by PBS. One hundred microliters of SureBlue TMB Microwell Peroxidase Substrate (VWR International, Radnor, PA, USA) was added to each well, and the plates were developed for 5–10 min. One hundred microliters of 1 N HCl was added to each well to stop the reaction, and the absorbance was read at 450 nm using a µQuant plate reader (Bio-Tek Instruments, Winooski, VT, USA). Treatment of the human breast cancer (MDA-MB-468) cells and Western blotting were performed using the methods described by us previously [22].

## 4. Conclusions

This article describes general synthetic routes to access novel symmetrical and cyclic marinopyrrole derivatives and evaluation of their *in vitro* activity against the binding of the pro-survival proteins, Mcl-1 and Bcl-x_L_, to the pro-apoptotic protein, Bim. The efforts were focused on improving anti-Mcl-1/Bim and Bcl-x_L_/Bim potency. The synthetic methods paved the way toward diverse sets of both symmetrical and cyclic marinopyrroles. SAR studies of marinopyrrole derivatives have clearly demonstrated that: (i) replacing the chlorines with bromines within the bispyrrole core improved the potency by two-fold (**1** *vs.* **9**); (ii) symmetrical marinopyrroles with substituents in the *para-*position to the carbonyl group are more potent against Mcl-1/Bim than Bcl-x_L_/Bim (Figure 2); (iii) the same trend was observed for cyclic marinopyrroles (Figure 3); (iv) cyclic marinopyrrole **3** is six- and seven-fold more potent than **1** against Mcl-1/Bim and Bcl-x_L_/Bim, respectively (Figure 3); (v) the cyclic marinopyrroles with certain substituents (OSO_2_SF_3_ and CO_2_Me) in the *para-*position to the carbonyl group are excellent “leads” for further optimization. In summary, we have designed and synthesized a series of novel symmetrical and cyclic marinopyrroles with improved potency against both Mcl-1 and Bcl-x_L_. Further optimization is actively ongoing, and the activity, selectivity and absorption, distribution, metabolism and excretion (ADME)/tox data of these compounds will be published in due course.

## Abbreviations

ADME: absorption, distribution, metabolism and excretion
calcd.: calculated
DCM: dichloromethane
dd: double doublet
*br*: broad
DPPP: bis(diphenylphosphino)propane
DIEA: diisopropylethylamine
DMF: dimethylformamide
DMSO: dimethyl sulfoxide
EtOAc: ethyl acetate
ESI: electrospray ionization
HPLC: high performance liquid chromatography
HPO(OEt)_2_: diethyl phosphanate
HRMS: high resolution mass spectrometry
HRP: horseradish peroxidase
IBX: 2-iodoxybenzoic acid
IR: infrared
KBr: potassium bromide
KF: potassium fluoride
LC: liquid chromatography
MeCN: acetonitrile
MeOH: methyl alcohol
MDA-MB-468: breast cancer cell line
mp: melting point
MRSA: methicillin-resistant *Staphylococcus aureus*
NCS: *N*-chlorosuccinimide
NaI: sodium iodide
NMR: nuclear magnetic resonance
PBS: phosphate-buffered saline
SAR: structure activity relationship
s: singlet
TBAF: tetrabutylammonium fluoride
TBDMS: *t*-butyldimethylsilyl
TBDMSCl: *t*-butyldimethylsilyl chloride
Tf: trifluoromethanesulfonyl
THF: tetrahydrofuran
TMB: 3,3′,5,5′-Tetramethylbenzidine
tox: toxicity

## Supplementary Files

Supplementary File 1 Supplementary Information (PDF, 1635 KB)
