# Supplementary material for: Cyclic Marinopyrrole Derivatives as Disruptors of Mcl-1 and Bcl-xL Binding to Bim
Source: Mar Drugs. 2014 Mar 7;12(3):1335–48. doi: 10.3390/md12031335 (PMC3967213; doi:10.3390/md12031335)
Supplement: Supplementary File 1 — Supplementary Information (PDF, 1635 KB) [file marinedrugs-12-01335-s001.pdf]

# Supplementary Information


**Figure S1.** $^{1}$H NMR spectrum of **3**.
**Figure S2.** $^{13}$C NMR spectrum of **3**.
**Figure S3.** $^{1}$H NMR spectrum of **6**.
**Figure S4.** $^{13}$C NMR spectrum of **6**.
**Figure S5.** $^{1}$H NMR spectrum of **4**.
**Figure S6.** $^{13}$C NMR spectrum of **4**.
**Figure S7.** $^{1}$H NMR spectrum of **4a**.
**Figure S8.** $^{13}$C NMR spectrum of **4a**.
**Figure S9.** $^{1}$H NMR spectrum of **7**.
**Figure S10.** $^{13}$C NMR spectrum of **7**.
**Figure S11.** $^{1}$H NMR spectrum of **7a**.
**Figure S12.** $^{13}$C NMR spectrum of **7a**.
**Figure S13.** $^{1}$H NMR spectrum of **5**.
**Figure S14.** $^{13}$C NMR spectrum of **5**.
**Figure S15.** $^{1}$H NMR spectrum of **5a**.
**Figure S16.** $^{13}$C NMR spectrum of **5a**.
**Figure S17.** $^{1}$H NMR spectrum of **8**.
**Figure S18.** $^{13}$C NMR spectrum of **8**.
**Figure S19.** $^{1}$H NMR spectrum of **8a**.
**Figure S20.** $^{13}$C NMR spectrum of **8a**.
**Figure S21.** Purity of **3** by HPLC.
**Figure S22.** Purity of **6** by HPLC.
**Figure S23.** Purity of **4** by HPLC.
**Figure S24.** Purity of **4a** by HPLC.
**Figure S25.** Purity of **7** by HPLC.
**Figure S26.** Purity of **7a** by HPLC.
**Figure S27.** Purity of **5** by HPLC.
**Figure S28.** Purity of **5a** by HPLC.
**Figure S29.** Purity of **8** by HPLC.
**Figure S30.** Purity of **8a** by HPLC.

**Figure S1.** $^1$H NMR spectrum of **3**.

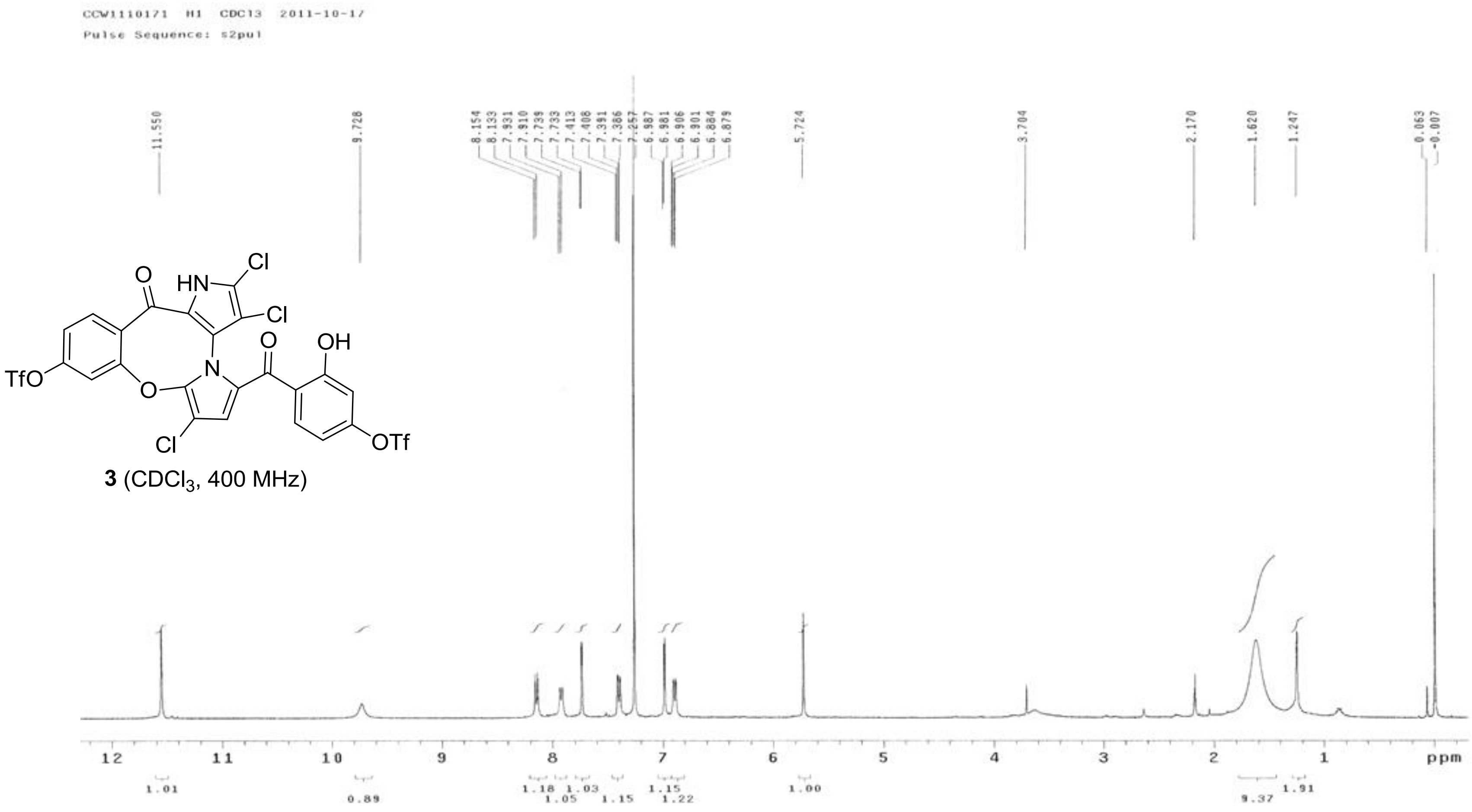

**Figure S2.** $^{13}$C NMR spectrum of **3**.

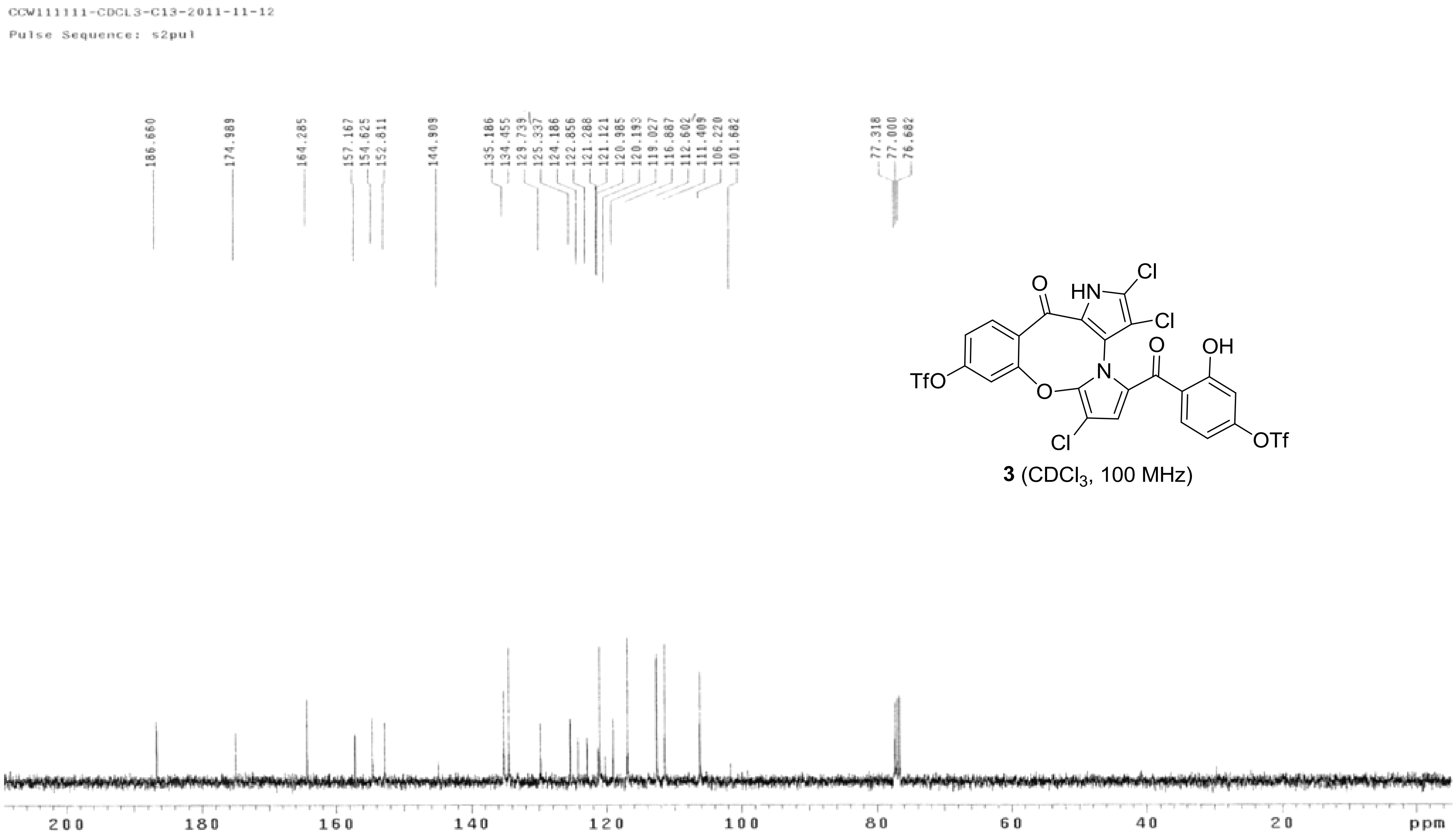

**Figure S3.** $^{1}$H NMR spectrum of **6**.

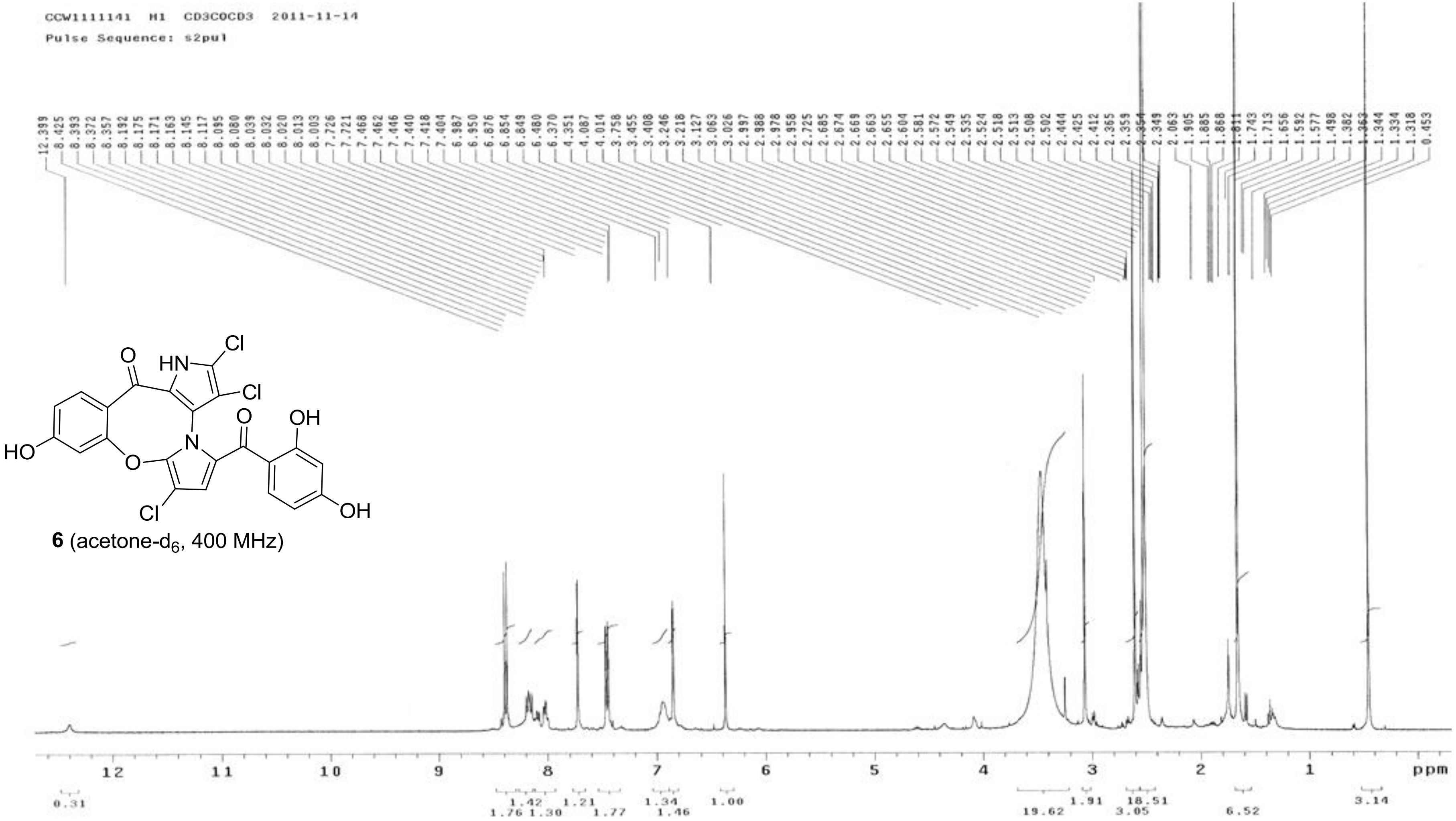

**Figure S4.** $^{13}$C NMR spectrum of **6**.

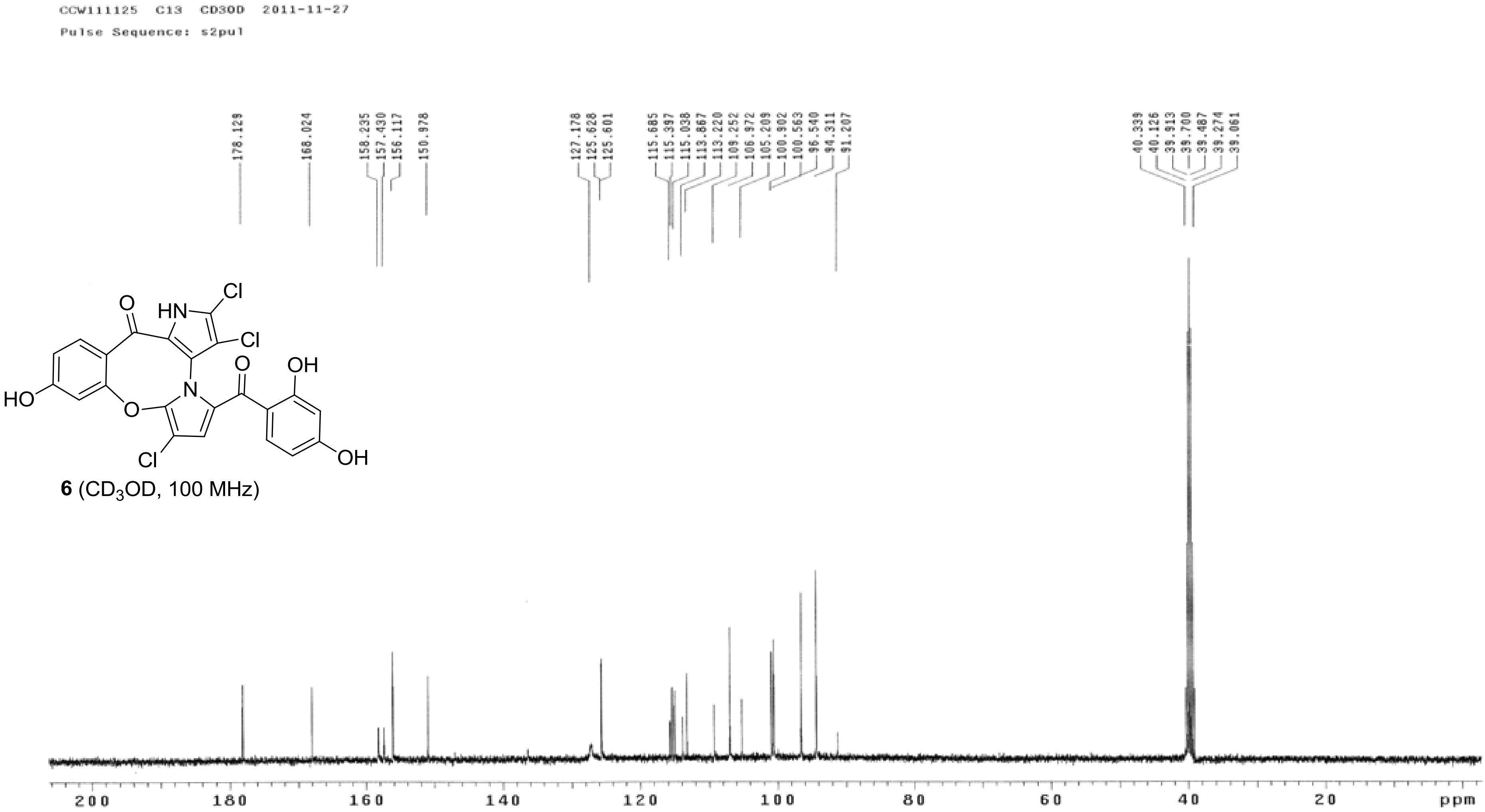

**Figure S5.** $^{1}$H NMR spectrum of **4**.

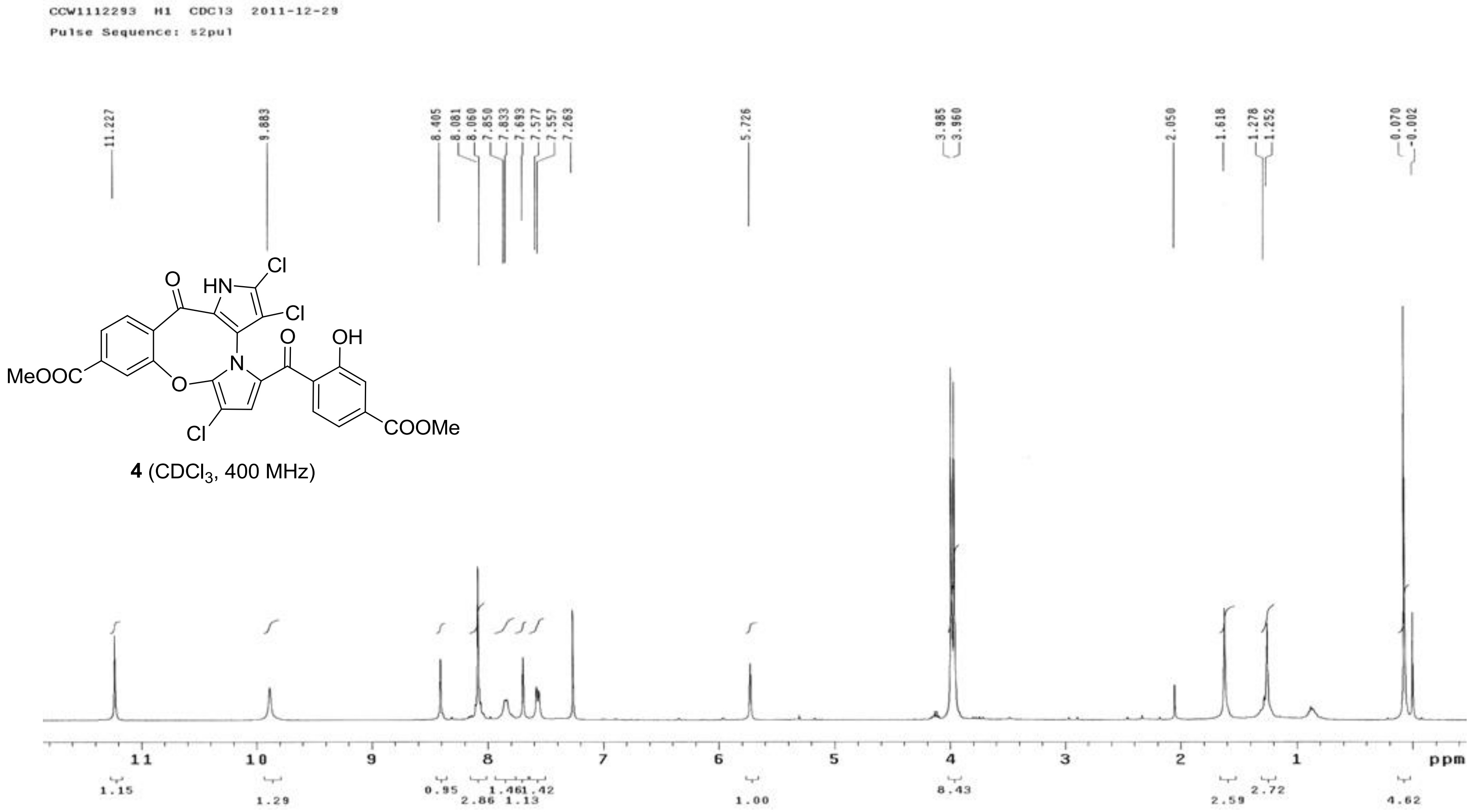

**Figure S6.** $^{13}$C NMR spectrum of **4**.

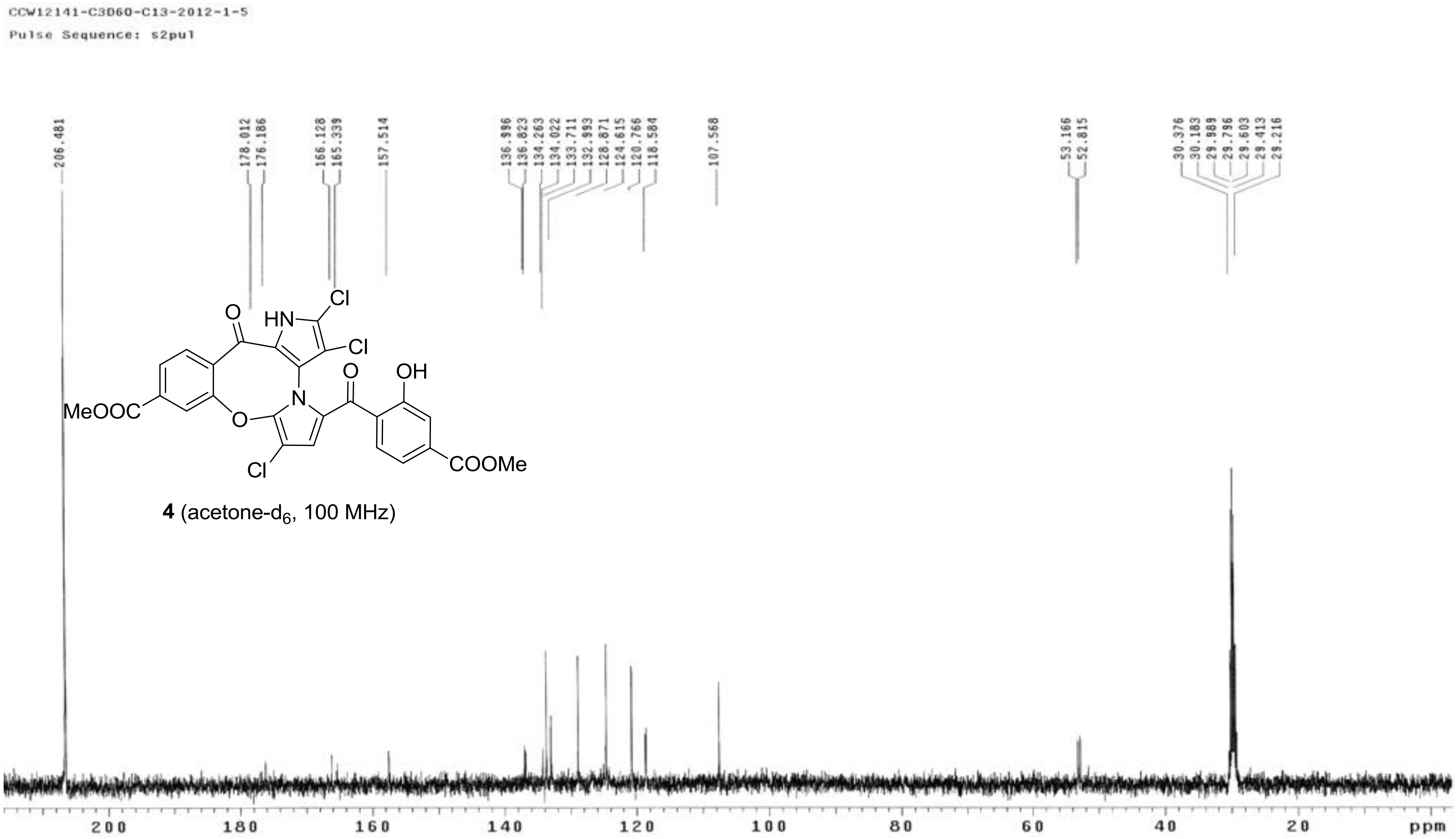

**Figure S7.** $^{1}$H NMR spectrum of **4a**.

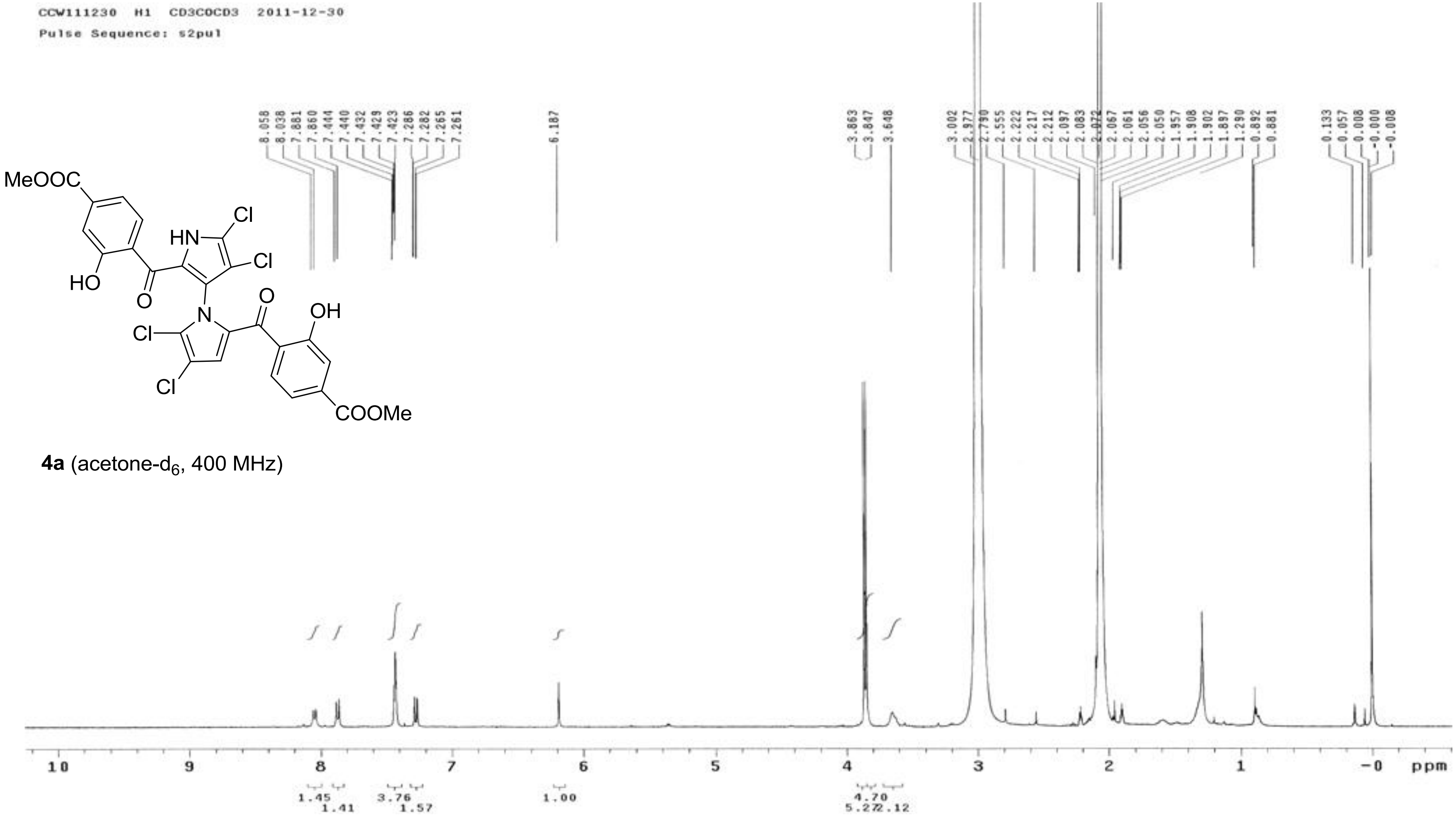

**Figure S8.** $^{13}$C NMR spectrum of **4a**.

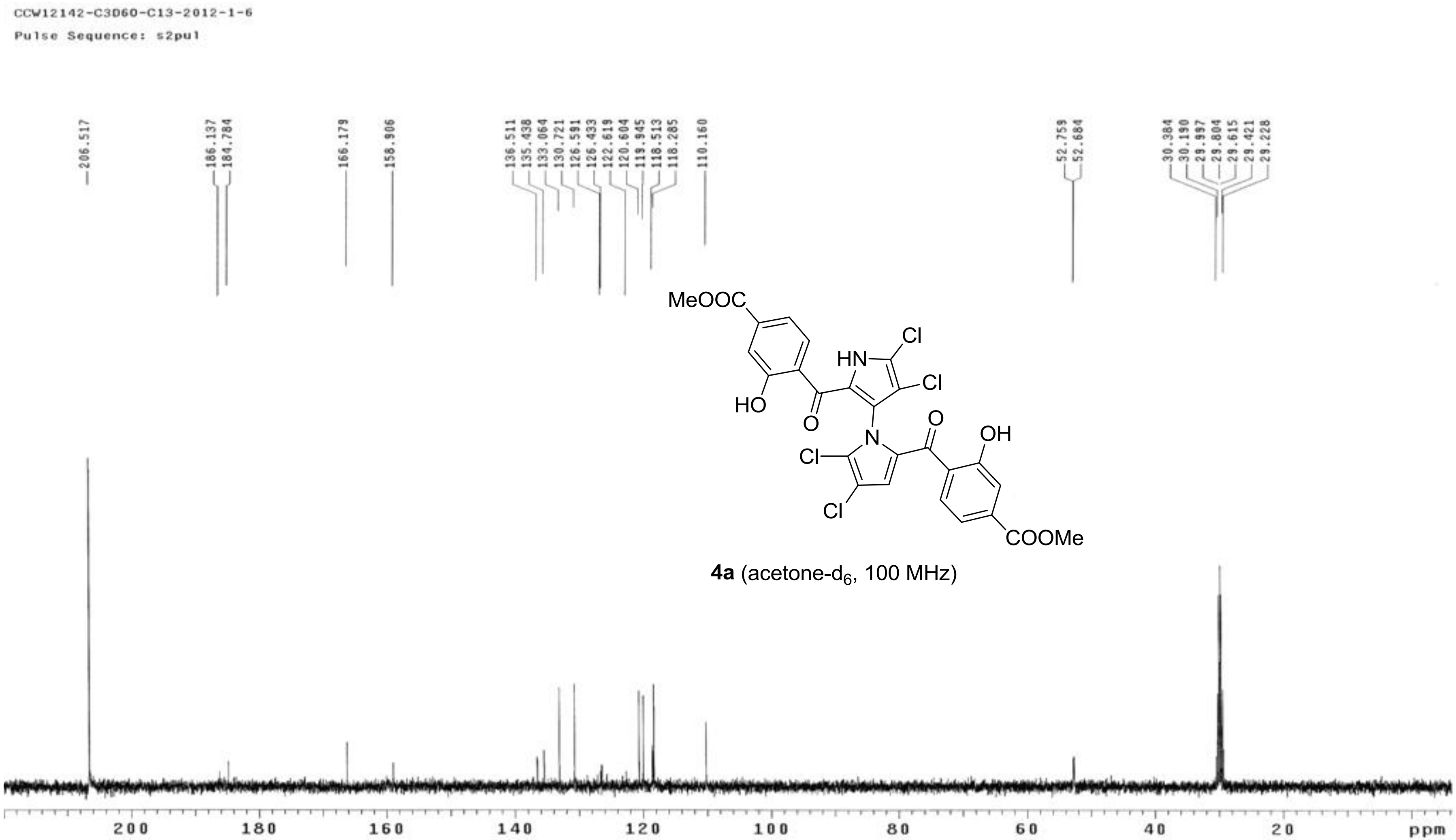

**Figure S9.** $^{1}$H NMR spectrum of **7**.

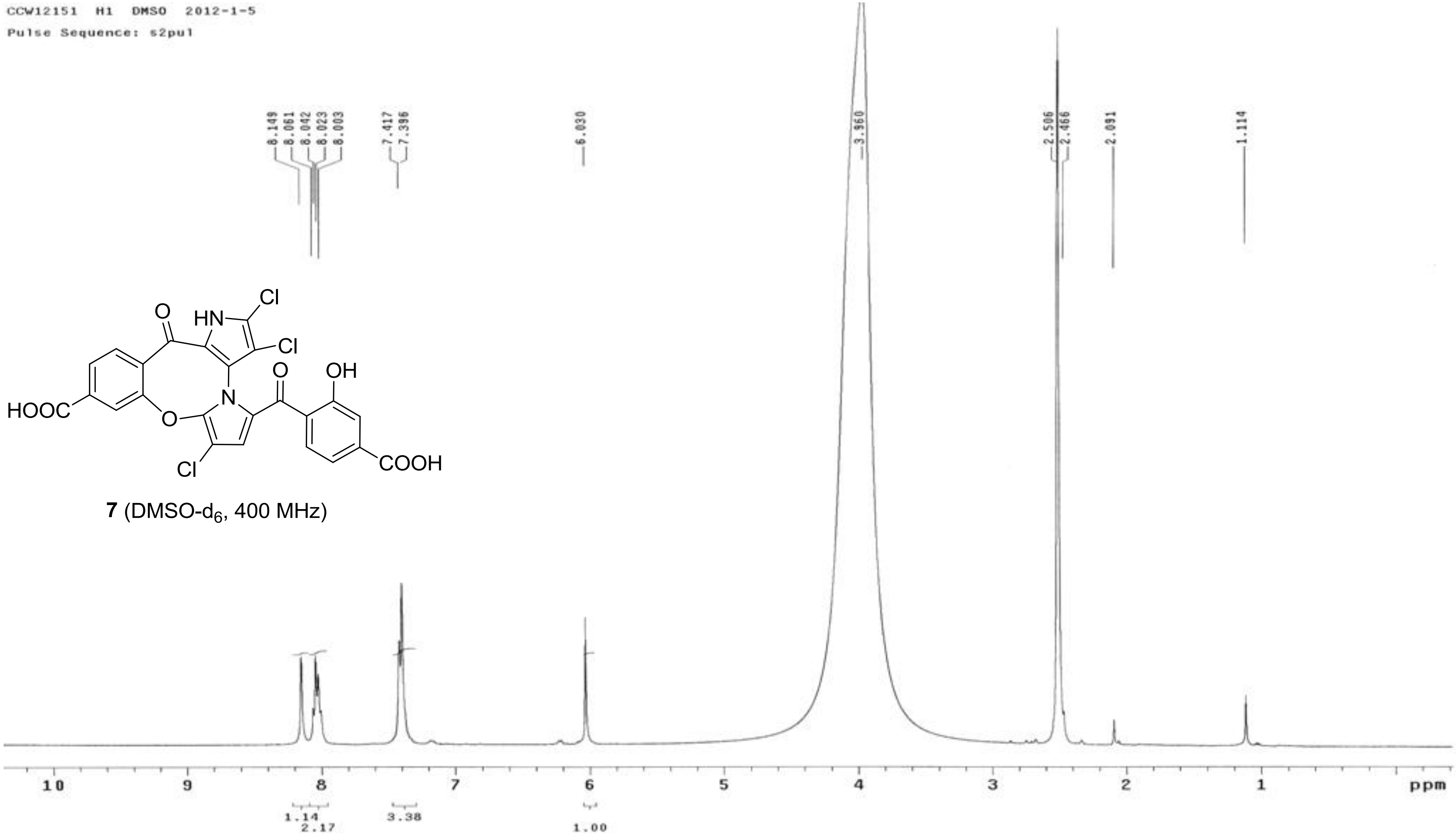

**Figure S10.** $^{13}$C NMR spectrum of **7**.

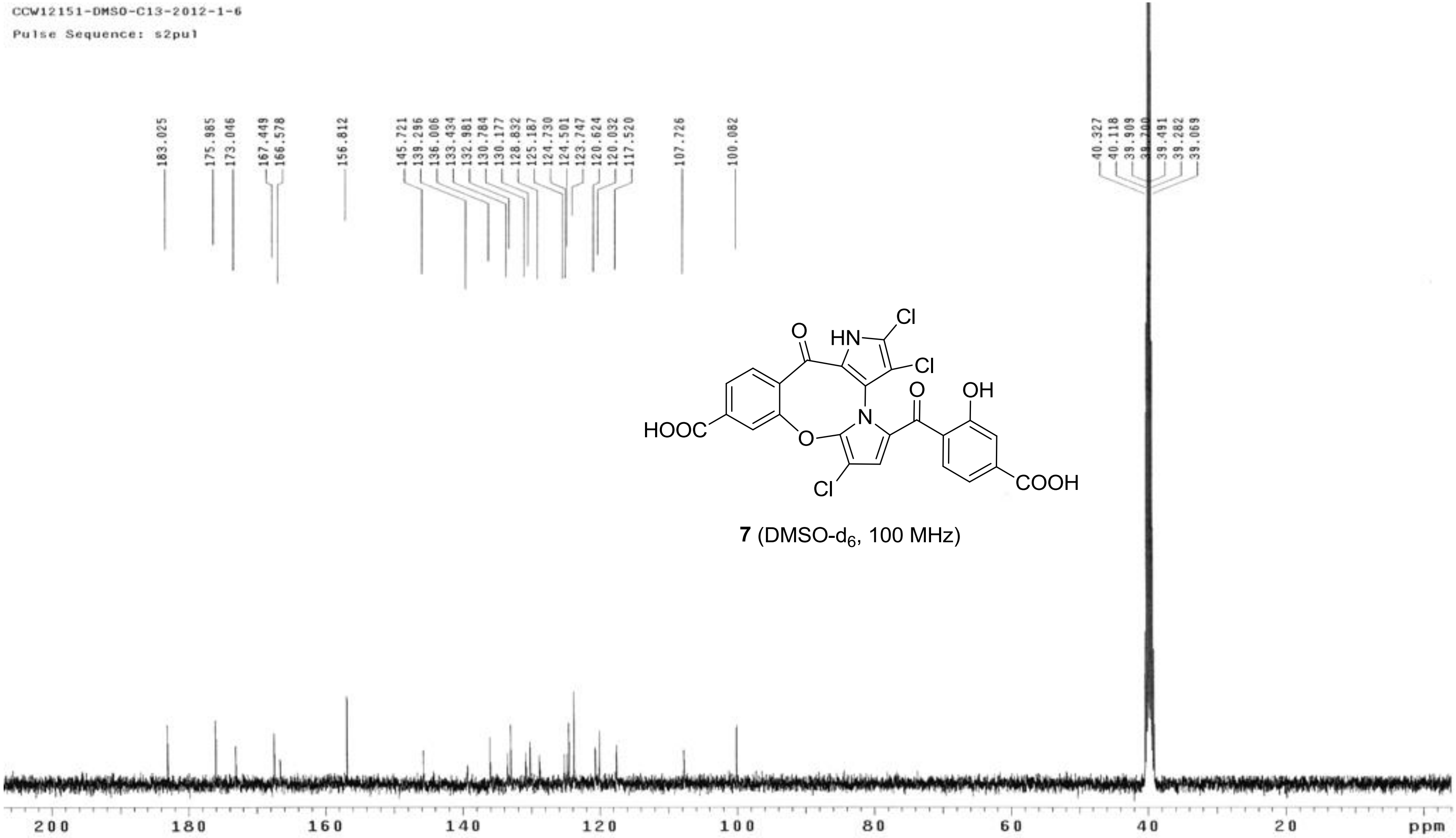

**Figure S11.** $^{1}$H NMR spectrum of **7a**.

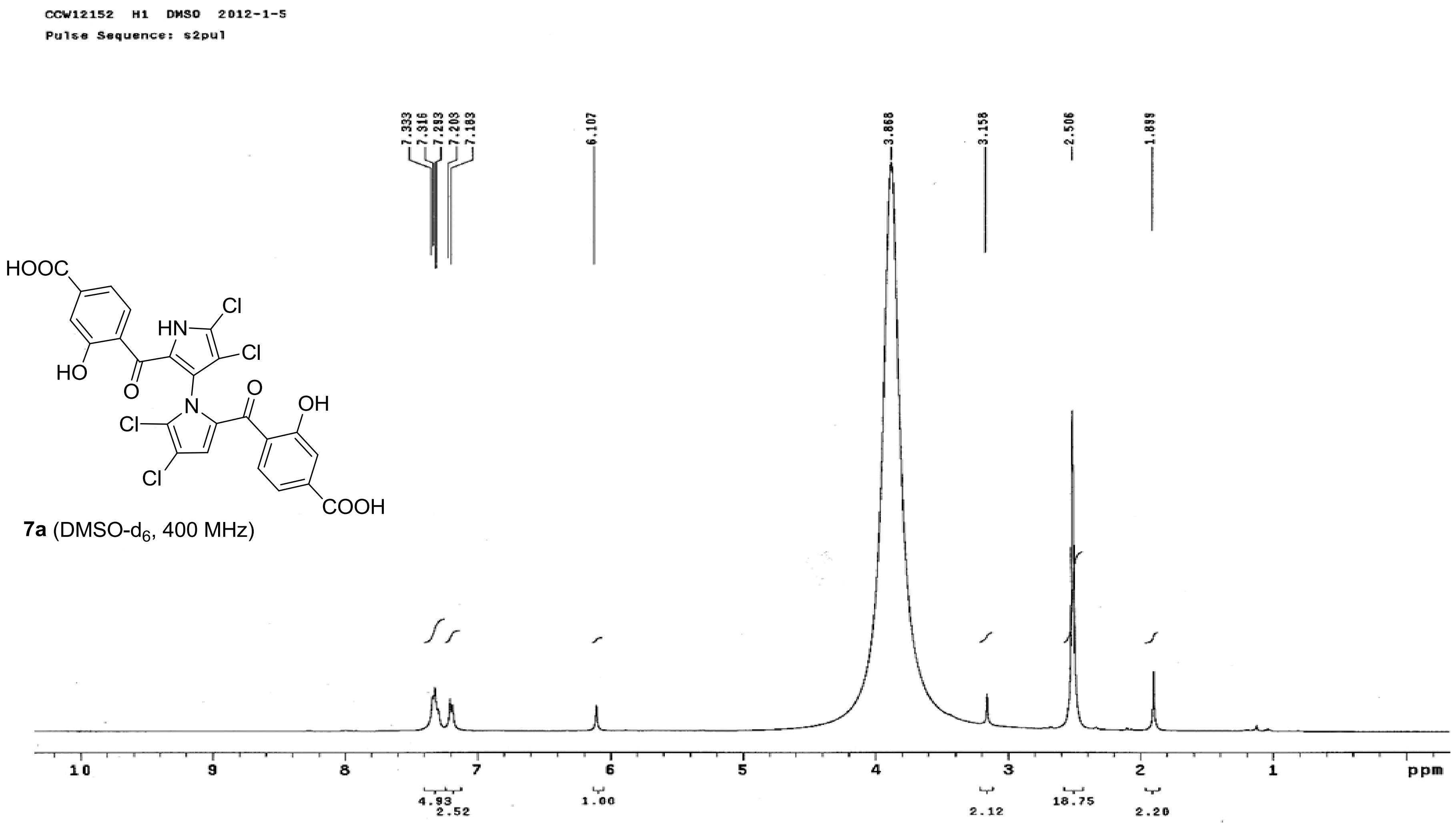

**Figure S12.** $^{13}C$ NMR spectrum of **7a**.

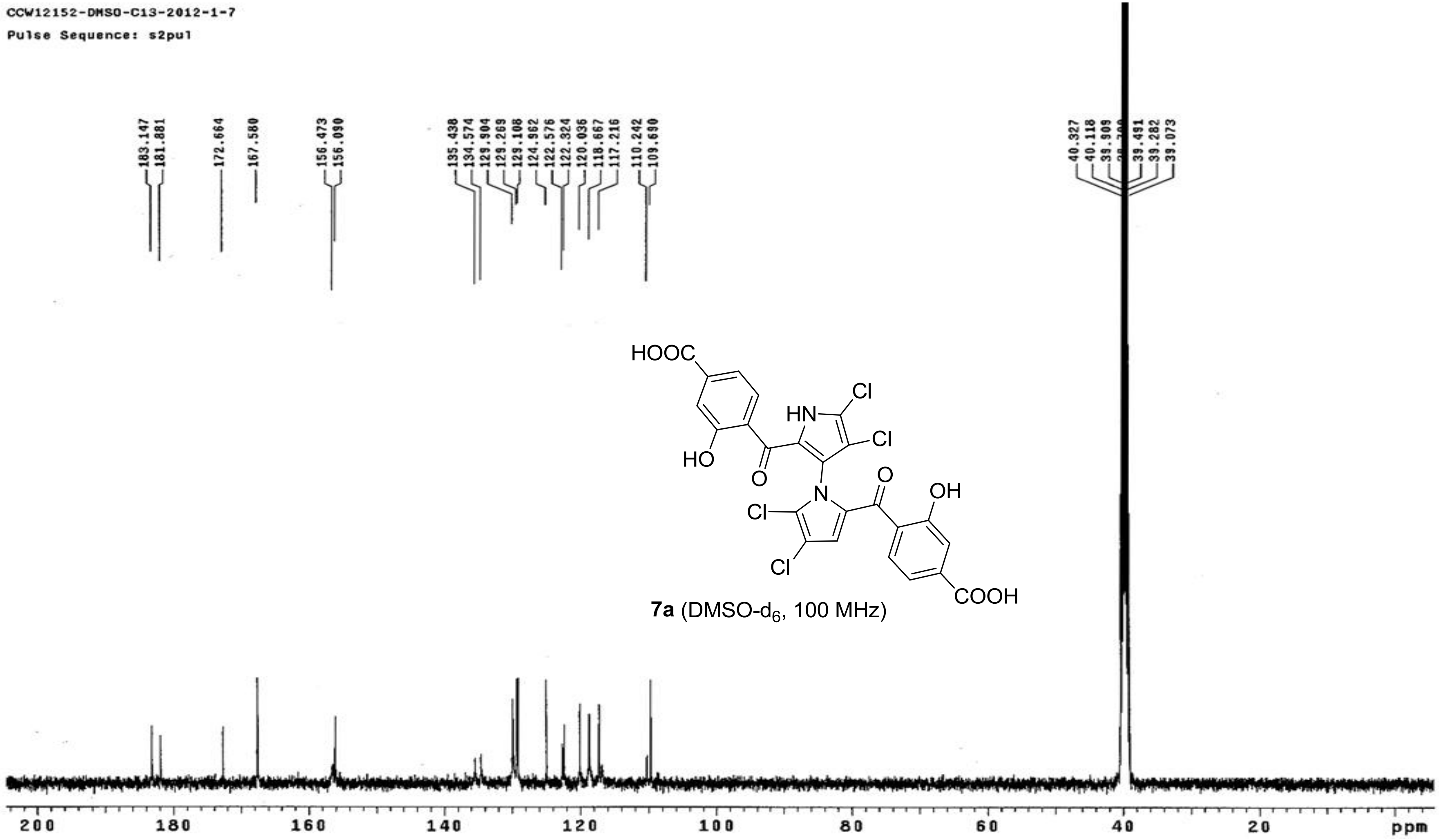

**Figure S13.** $^{1}$H NMR spectrum of **5**.

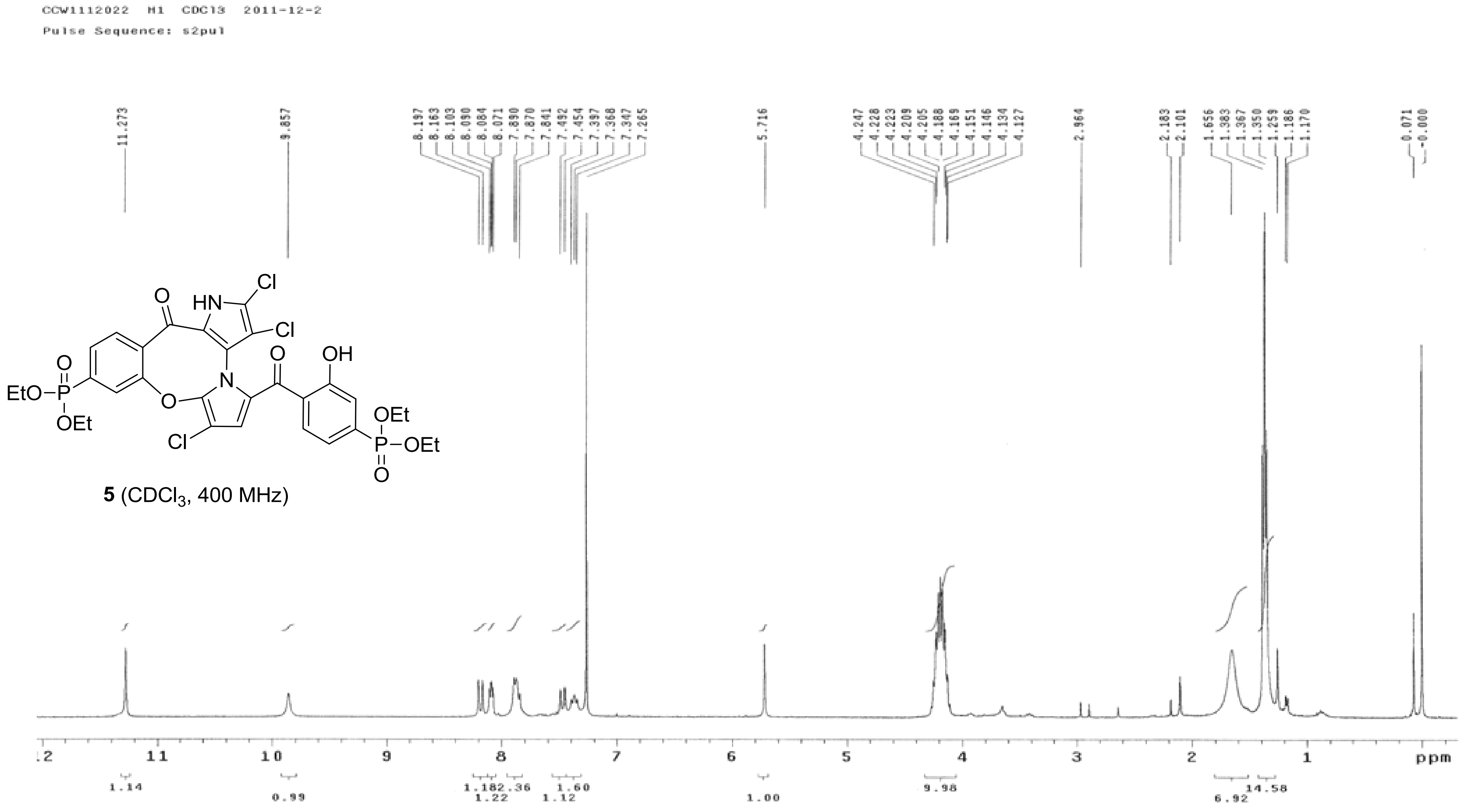

**Figure S14.** $^{13}C$ NMR spectrum of **5**.

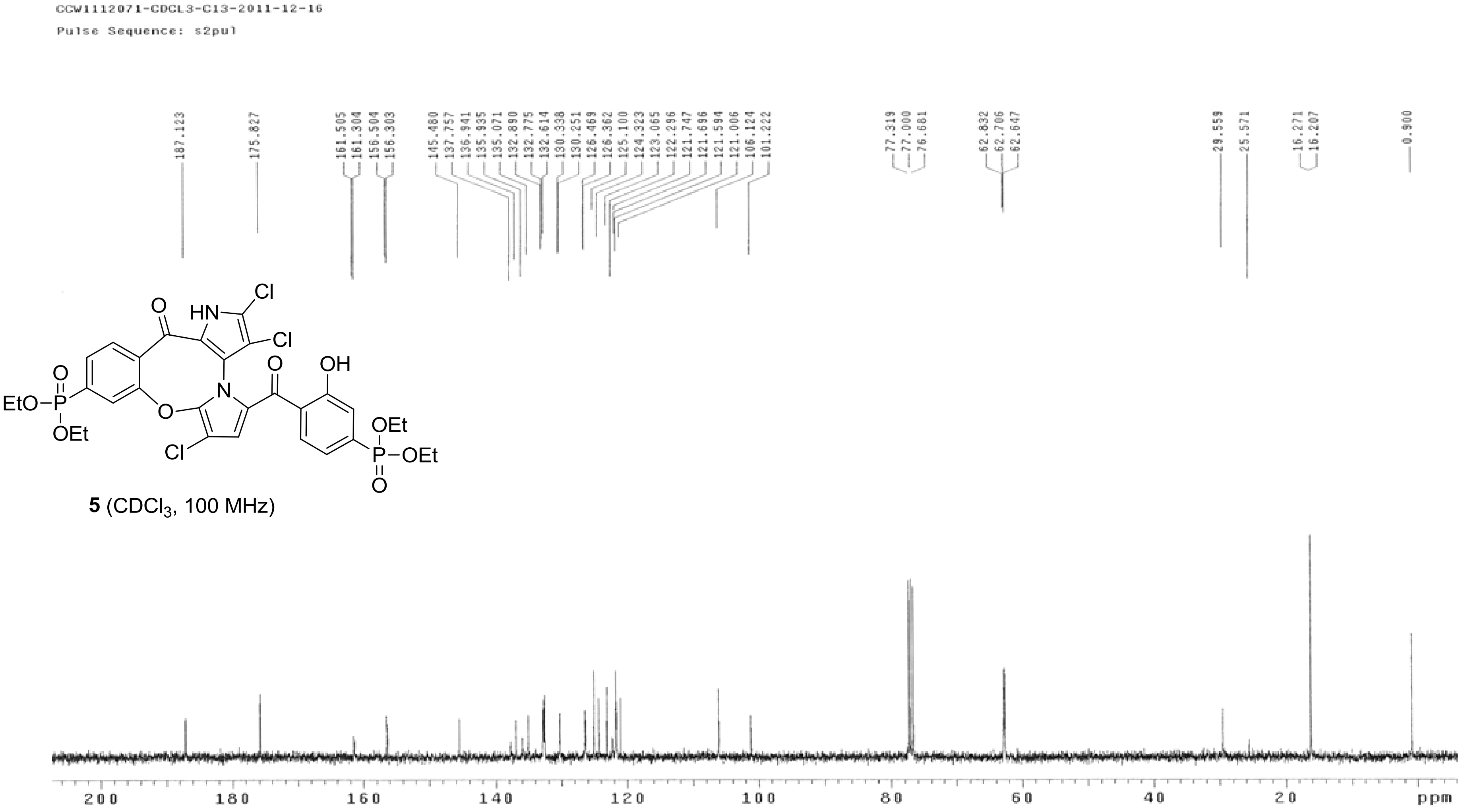

**Figure S15.** $^1$H NMR spectrum of **5a**.

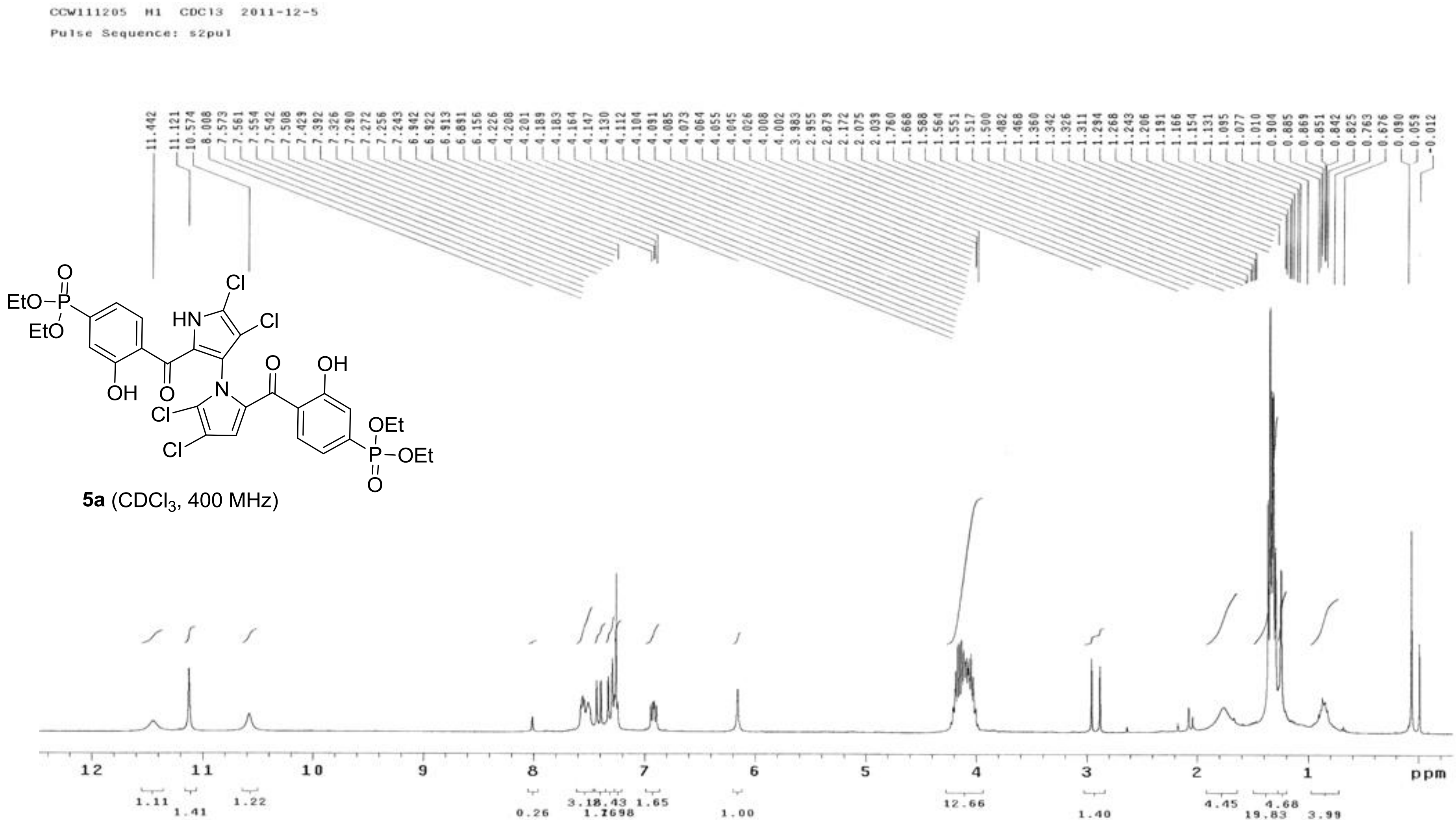

**Figure S16.** $^{13}C$ NMR spectrum of **5a**.

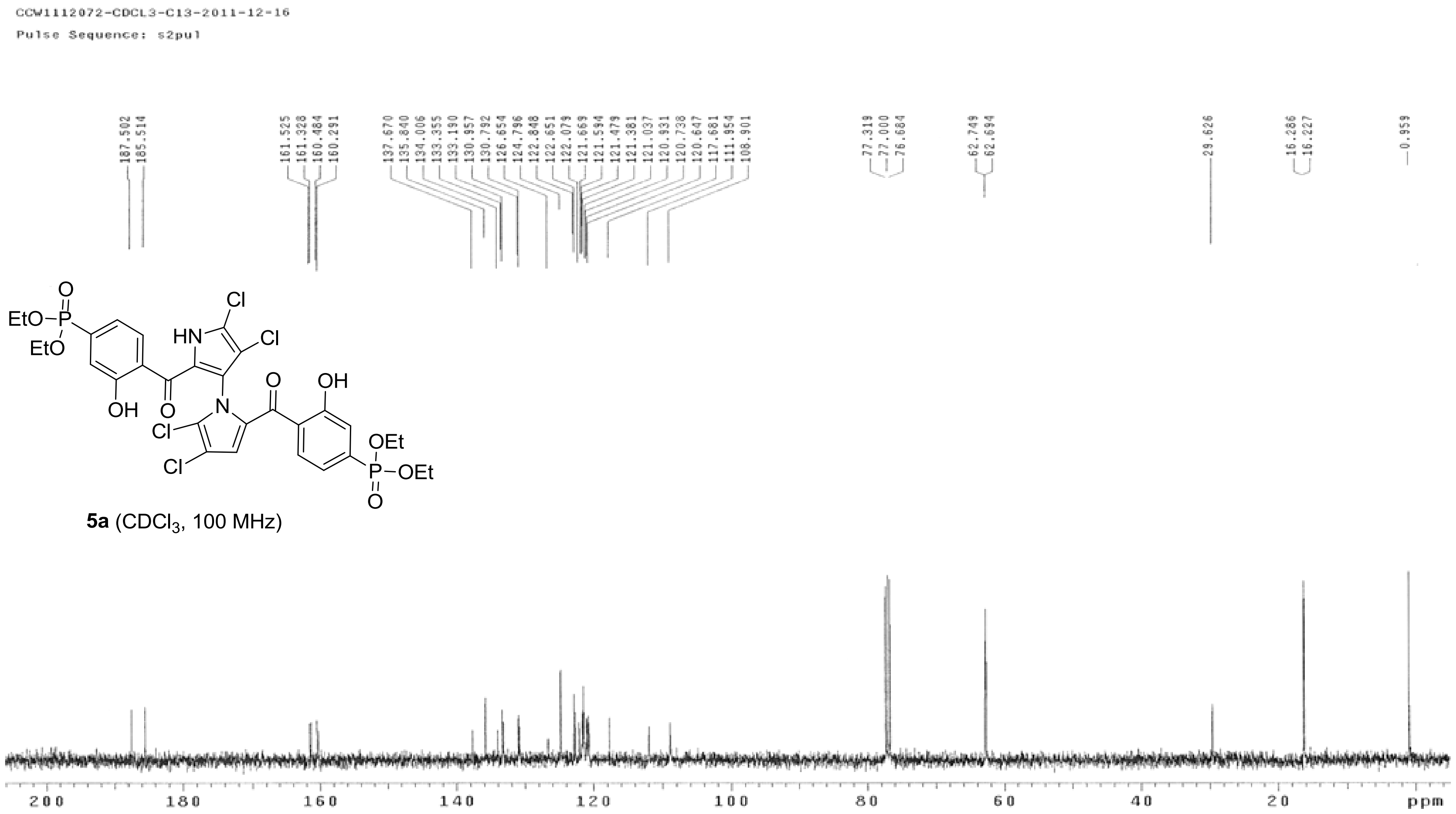

**Figure S17.** $^{1}$H NMR spectrum of **8**.

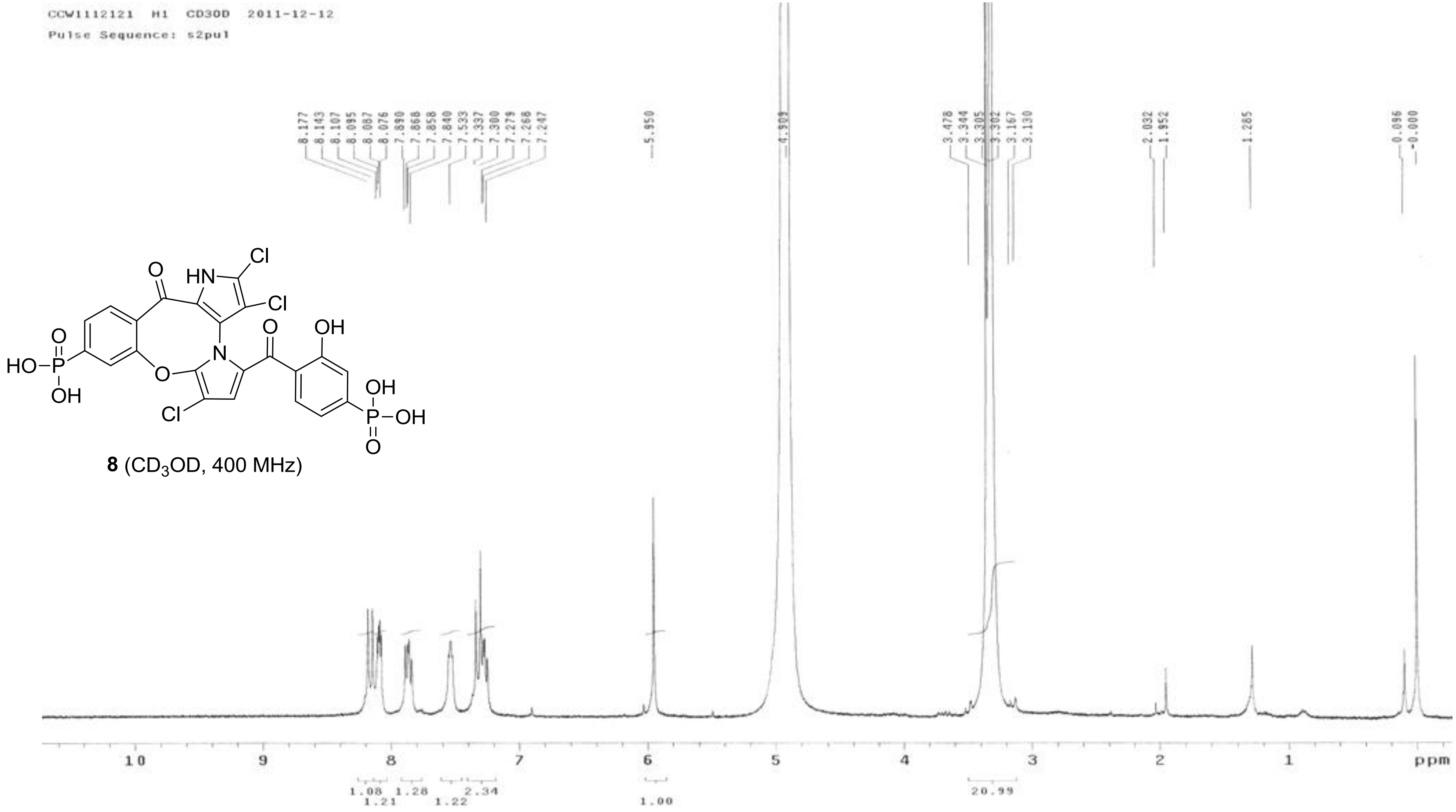

**Figure S18.** $^{13}C$ NMR spectrum of **8**.

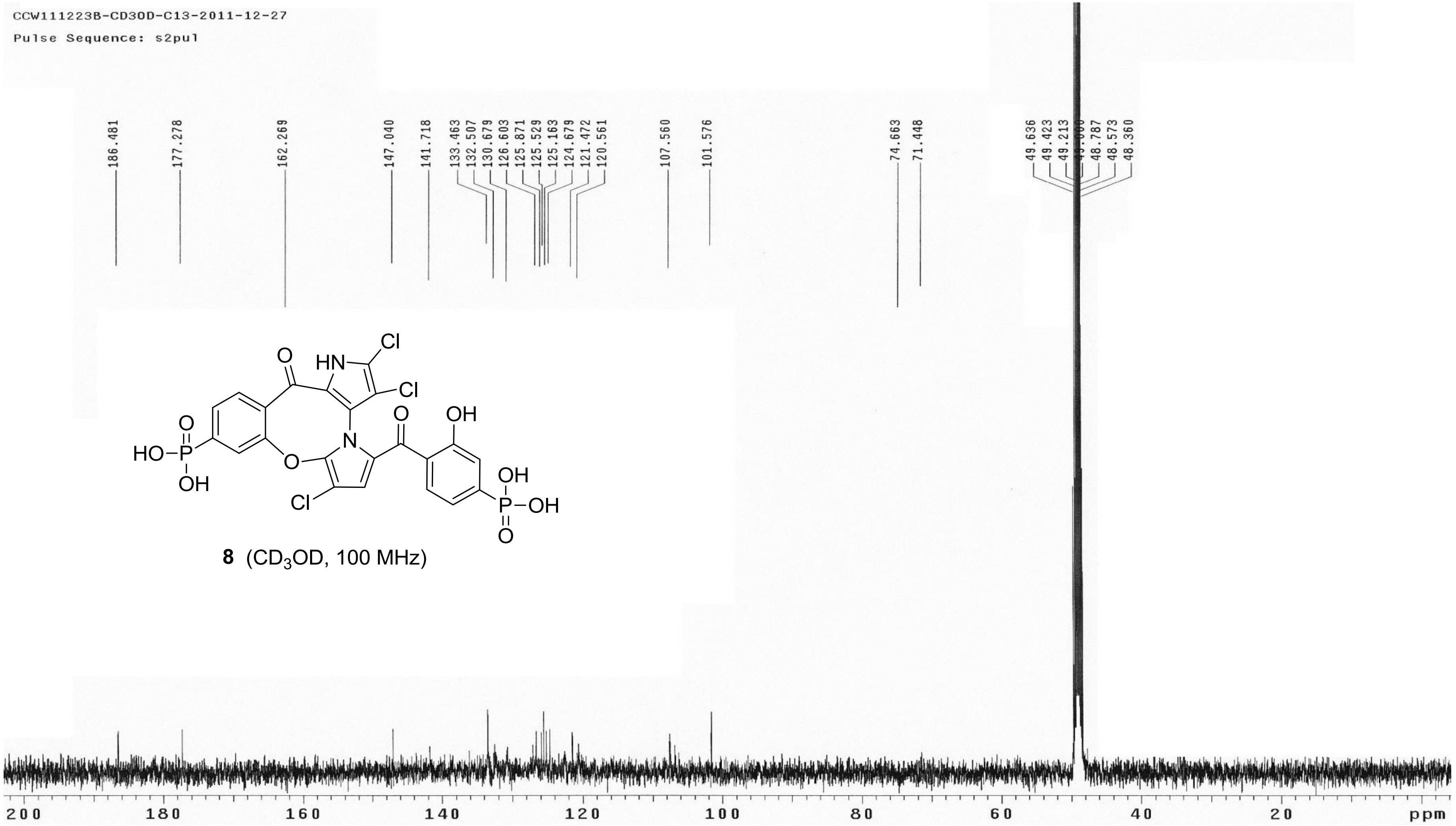

**Figure S19.** $^1$H NMR spectrum of **8a**.

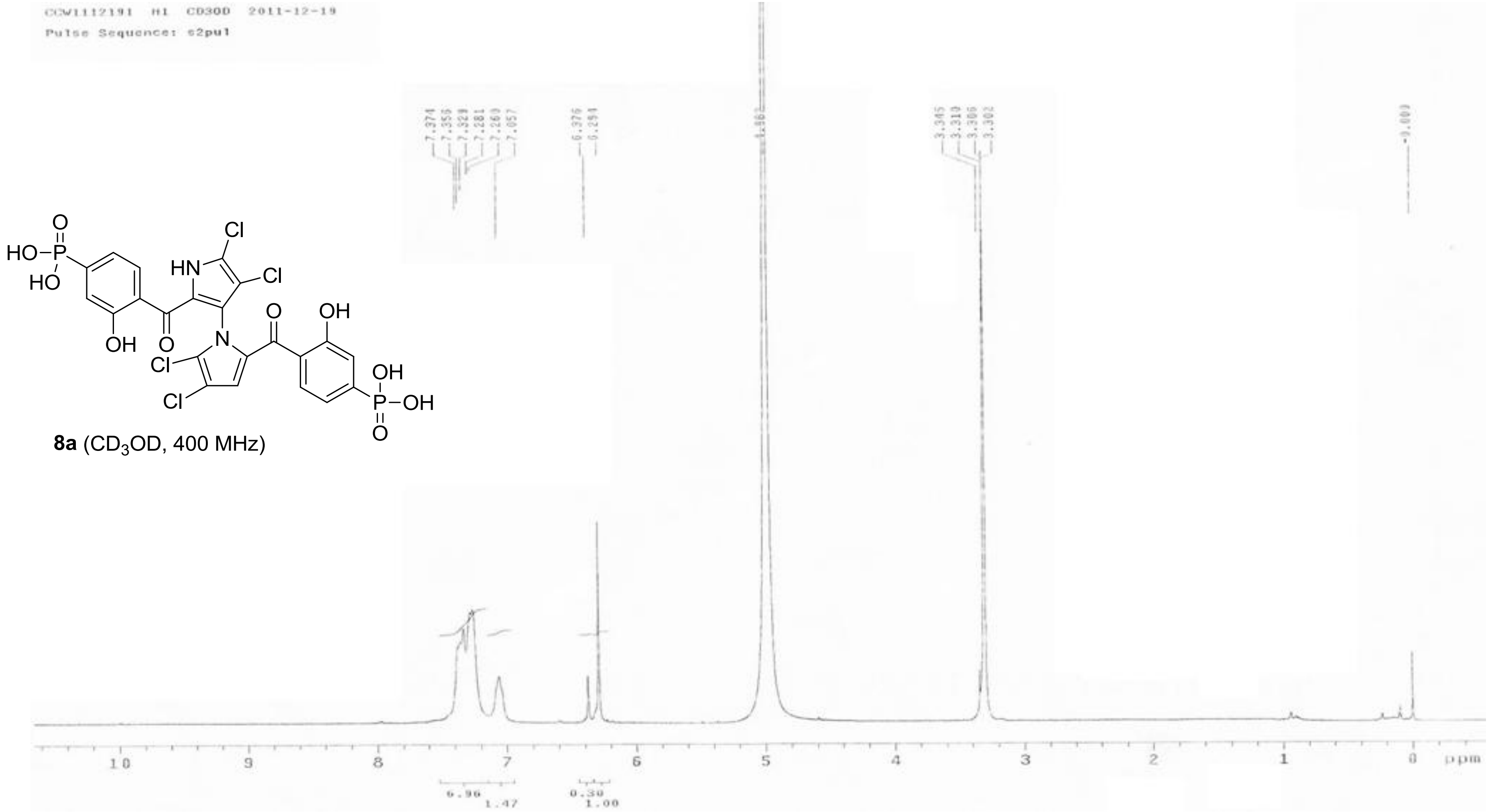

**Figure S20.** $^{13}$C NMR spectrum of **8a**.

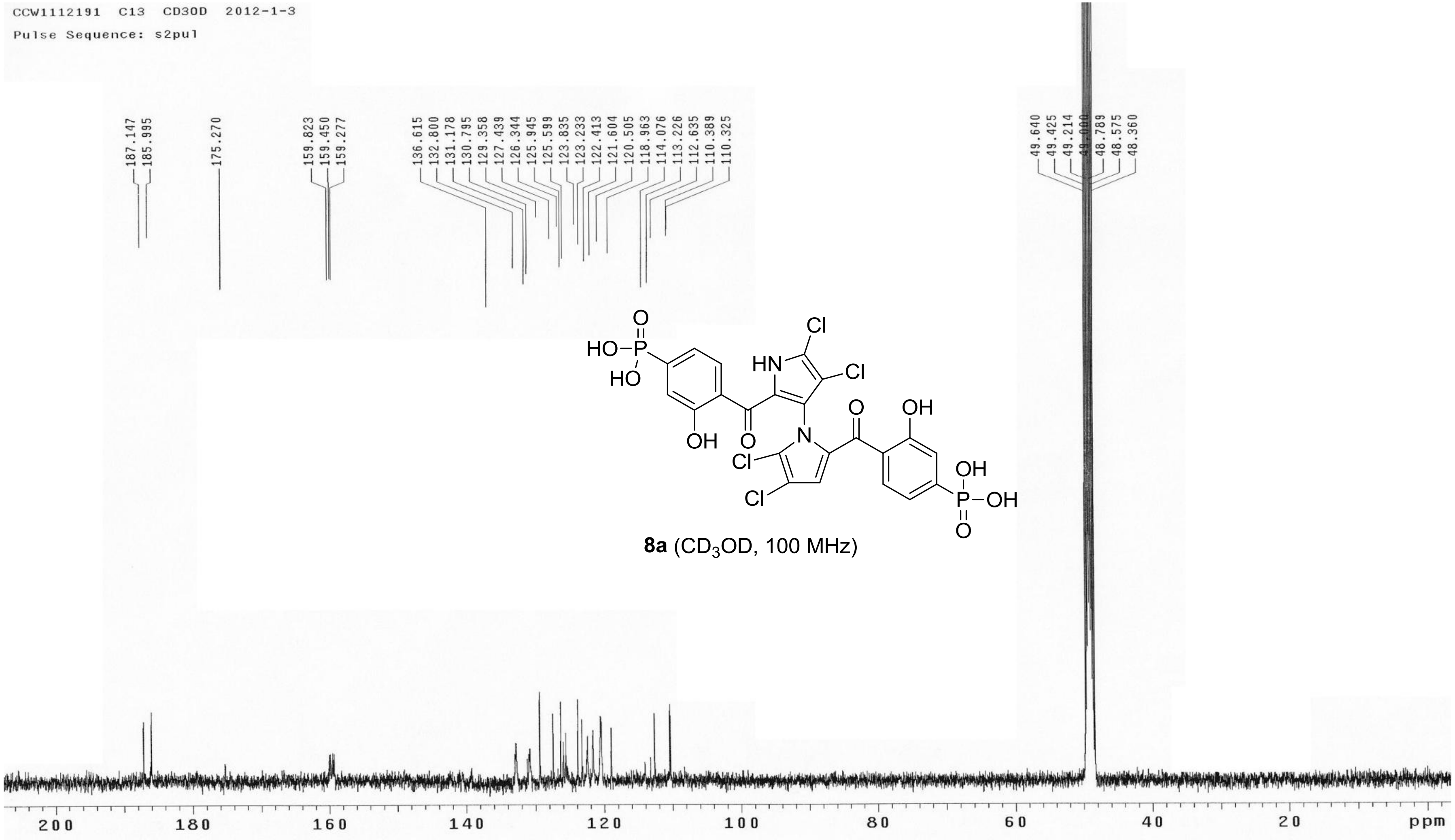

**Figure S21.** Purity of **3** by HPLC.

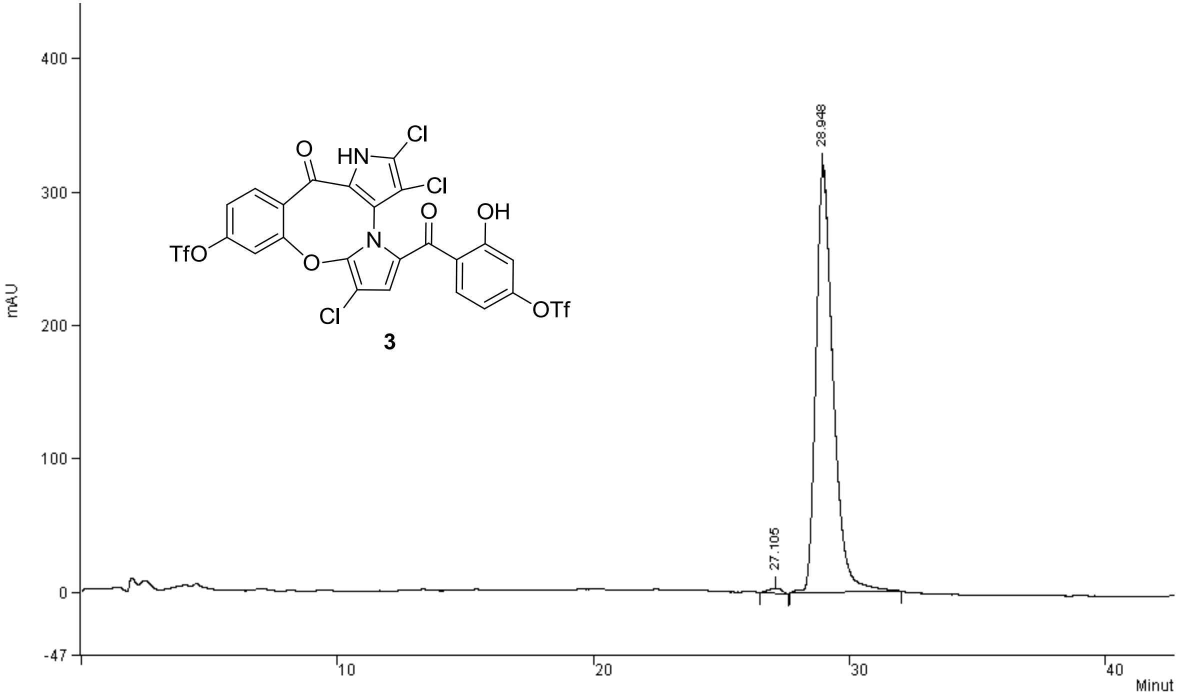


| Peak No. | Peak Name | Result () | Ret. Time (min) | Time Offset (min) | Area (counts) | Sep. Code | Width 1/2 (sec) |
|---|---|---|---|---|---|---|---|
| 1 | | 0.8561 | 27.105 | 0.000 | 1295006 | BB | 31.6 |
| 2 | | 99.1439 | 28.948 | 0.000 | 149970096 | BB | 41.3 |
| | Totals: | 100.0000 | | 0.000 | 151265102 | | |

**Figure S22.** Purity of **6** by HPLC.

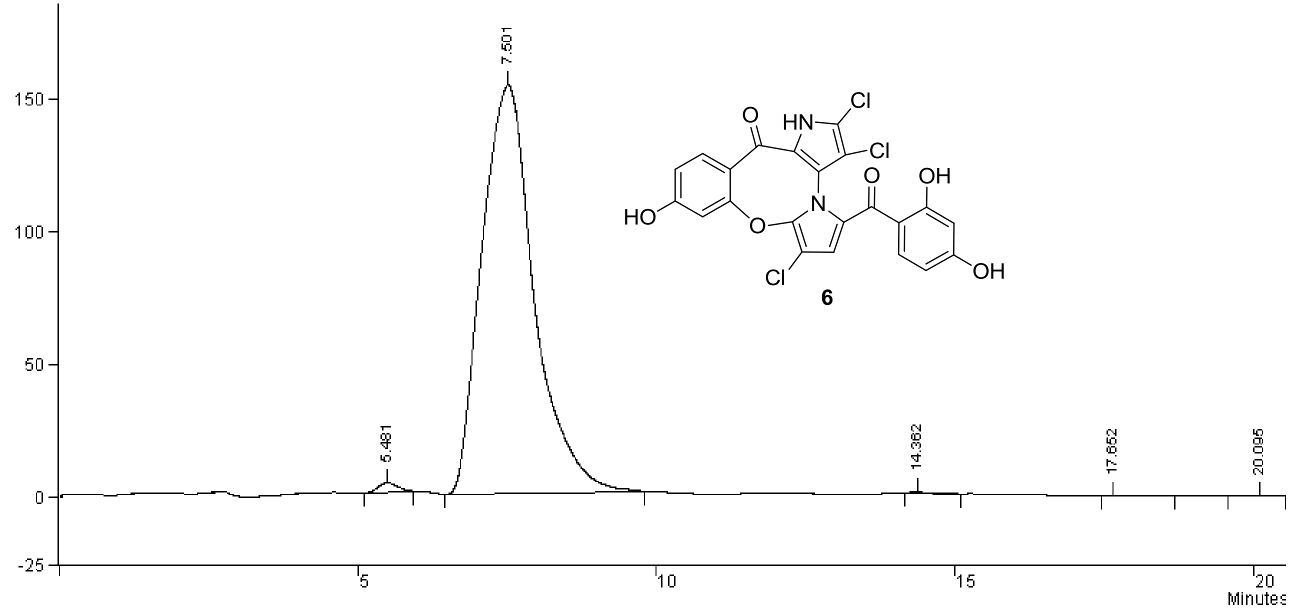


| Peak No. | Peak Name | Result () | Ret. Time (min) | Time Offset (min) | Area (counts) | Sep. Code | Width 1/2 (sec) |
|---|---|---|---|---|---|---|---|
| 1 | | 0.7887 | 5.481 | 0.000 | 748973 | BB | 23.3 |
| 2 | | 98.8856 | 7.501 | 0.000 | 93902016 | BB | 56.6 |
| 3 | | 0.1127 | 14.362 | 0.000 | 107065 | BB | 0.0 |
| 4 | | 0.0884 | 17.652 | 0.000 | 83987 | BB | 0.0 |
| 5 | | 0.1245 | 20.095 | 0.000 | 118235 | BB | 41.9 |
| | Totals: | 99.9999 | | 0.000 | 94960276 | | |

**Figure S23.** Purity of **4** by HPLC.

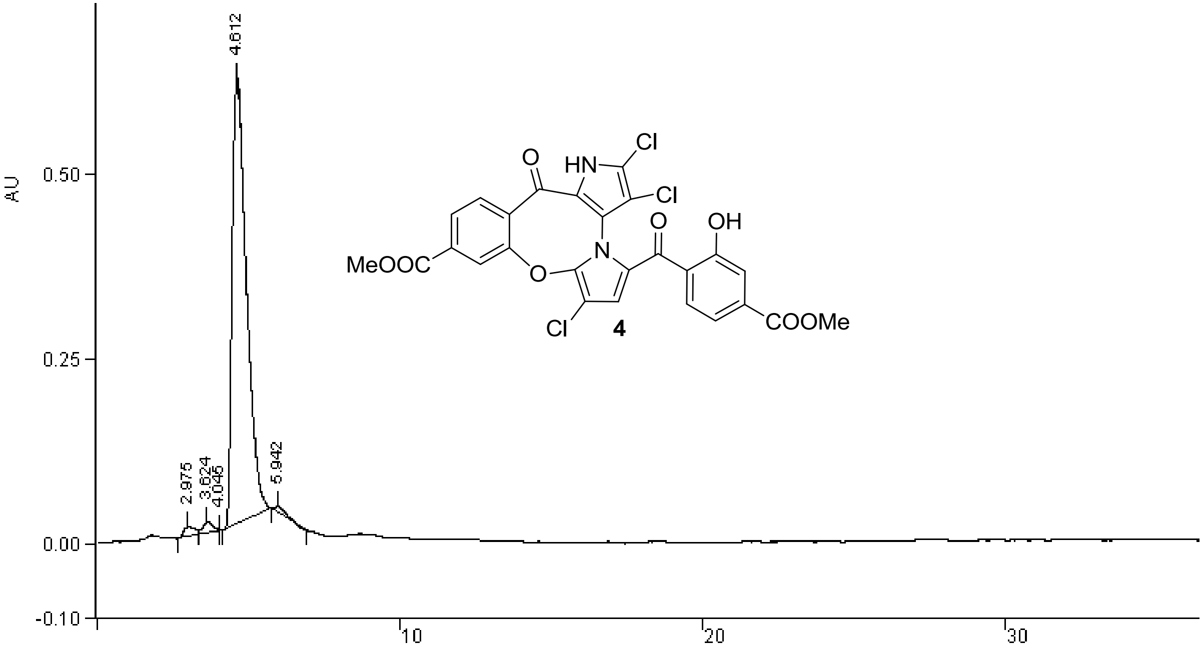


| Peak No. | Peak Name | Result () | Ret. Time (min) | Time Offset (min) | Area (counts) | Sep. Code | Width 1/2 (sec) |
|---|---|---|---|---|---|---|---|
| 1 | | 1.6994 | 2.975 | 0.000 | 3522723 | BV | 0.0 |
| 2 | | 1.8720 | 3.624 | 0.000 | 3880466 | VV | 0.0 |
| 3 | | 0.0228 | 4.045 | 0.000 | 47190 | VP | 0.0 |
| 4 | | 95.6192 | 4.612 | 0.000 | 198208016 | PB | 31.9 |
| 5 | | 0.7866 | 5.942 | 0.000 | 1630602 | BB | 10.1 |
| | Totals: | 100.0000 | | 0.000 | 207288997 | | |

**Figure S24.** Purity of **4a** by HPLC.

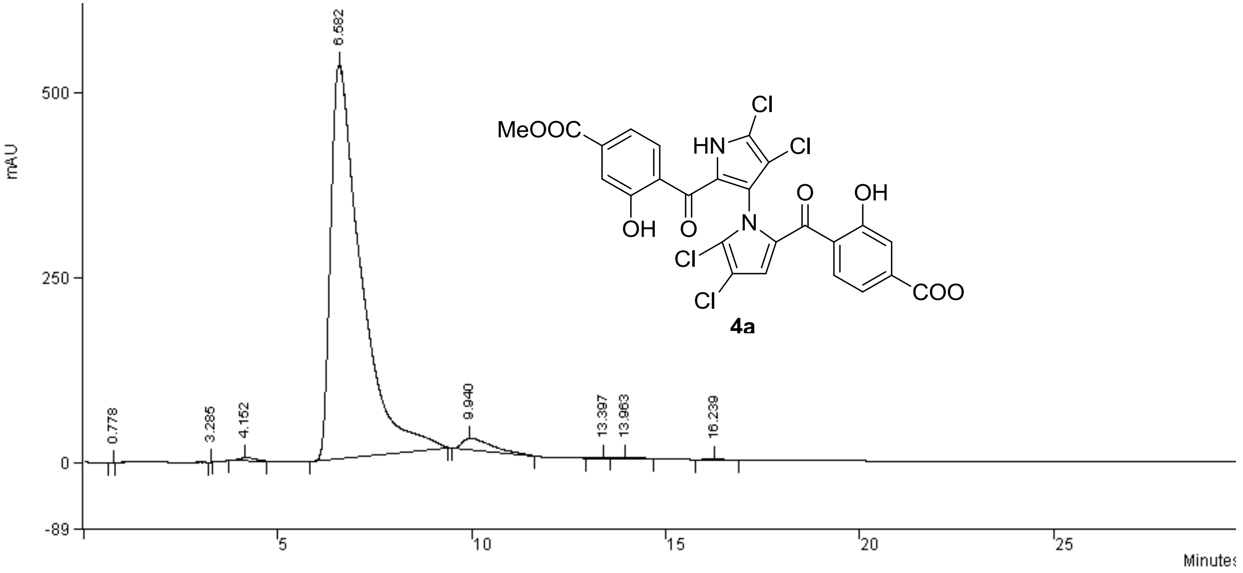


| Peak No. | Peak Name | Result () | Ret. Time (min) | Time Offset (min) | Area (counts) | Sep. Code | Width 1/2 (sec) |
|---|---|---|---|---|---|---|---|
| 1 | | 0.0056 | 0.778 | 0.000 | 16843 | BB | 0.0 |
| 2 | | 0.0031 | 3.285 | 0.000 | 9358 | BB | 0.0 |
| 3 | | 0.3875 | 4.152 | 0.000 | 1164280 | BB | 23.9 |
| 4 | | 96.2882 | 6.582 | 0.000 | 289314240 | BB | 46.8 |
| 5 | | 2.9043 | 9.940 | 0.000 | 8726362 | BB | 36.9 |
| 6 | | 0.0837 | 13.397 | 0.000 | 251561 | BV | 26.4 |
| 7 | | 0.1909 | 13.963 | 0.000 | 573666 | VB | 70.8 |
| 8 | | 0.1367 | 16.239 | 0.000 | 410600 | BB | 32.5 |
| | Totals: | 100.0000 | | 0.000 | 300466910 | | |

**Figure S25.** Purity of **7** by HPLC.

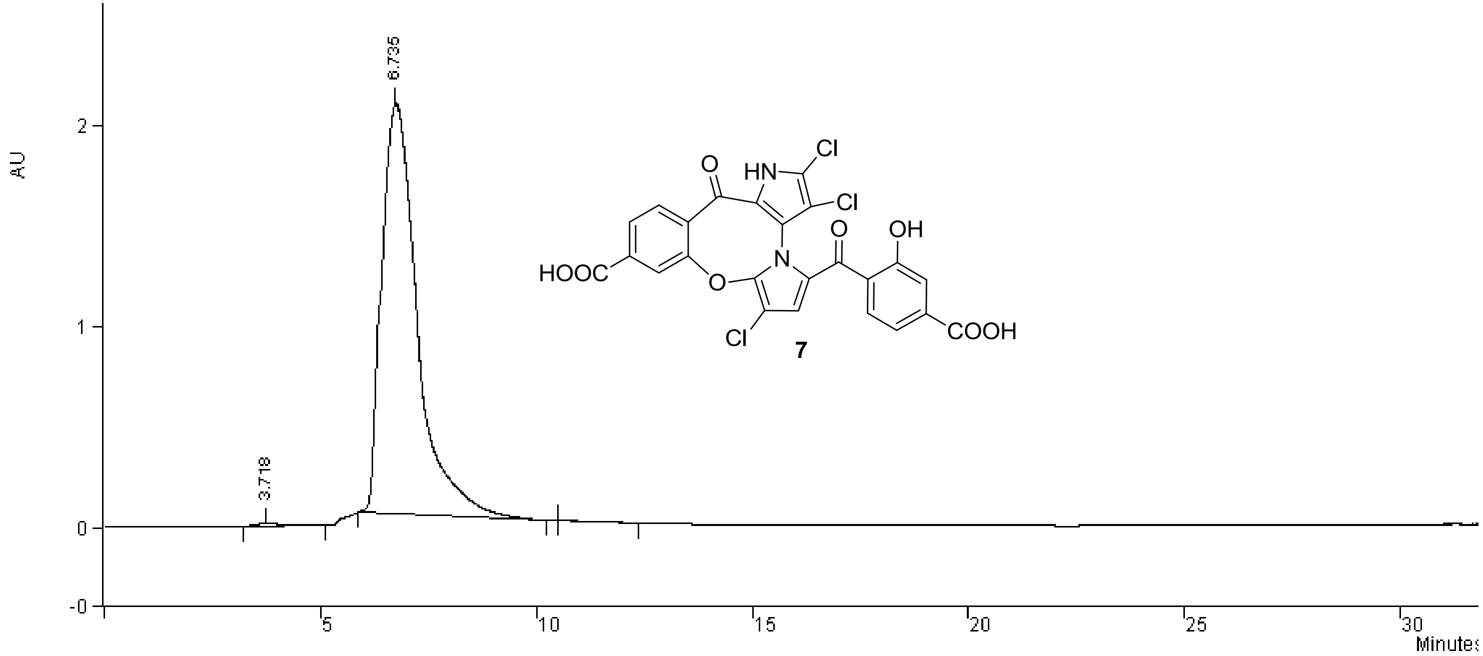


| Peak No. | Peak Name | Result () | Ret. Time (min) | Time Offset (min) | Area (counts) | Sep. Code | Width 1/2 (sec) |
|---|---|---|---|---|---|---|---|
| 1 | | 0.4585 | 3.718 | 0.000 | 5404698 | BB | 54.2 |
| 2 | | 99.3966 | 6.735 | 0.000 | 1171587968 | BB | 51.8 |
| 3 | | 0.1449 | 10.498 | 0.000 | 1707659 | BB | 0.0 |
| | Totals: | 100.0000 | | 0.000 | 1178700325 | | |

**Figure S26.** Purity of **7a** by HPLC.

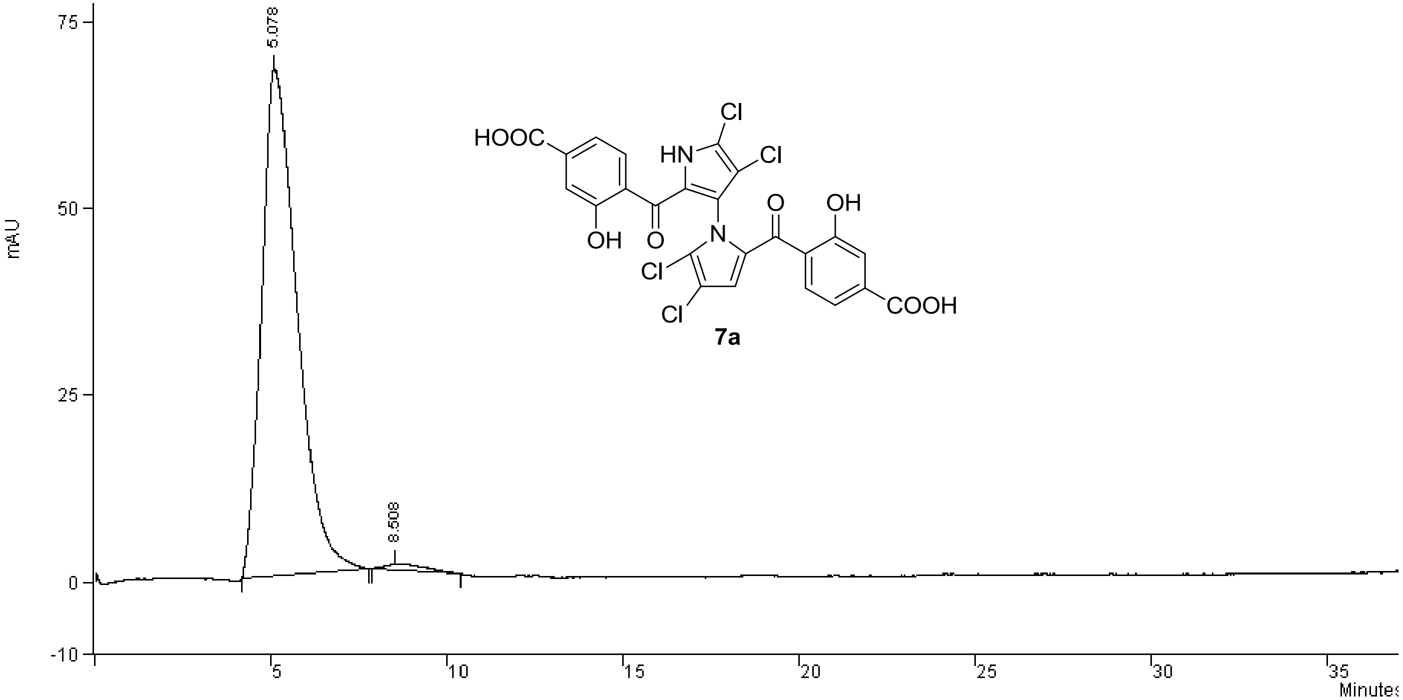


| Peak No. | Peak Name | Result () | Ret. Time (min) | Time Offset (min) | Area (counts) | Sep. Code | Width 1/2 (sec) |
|---|---|---|---|---|---|---|---|
| 1 | | 98.6235 | 5.078 | 0.000 | 47575300 | BB | 63.9 |
| 2 | | 1.3765 | 8.508 | 0.000 | 664007 | BB | 55.7 |
| | Totals: | 100.0000 | | 0.000 | 48239307 | | |

**Figure S27.** Purity of **5** by HPLC.

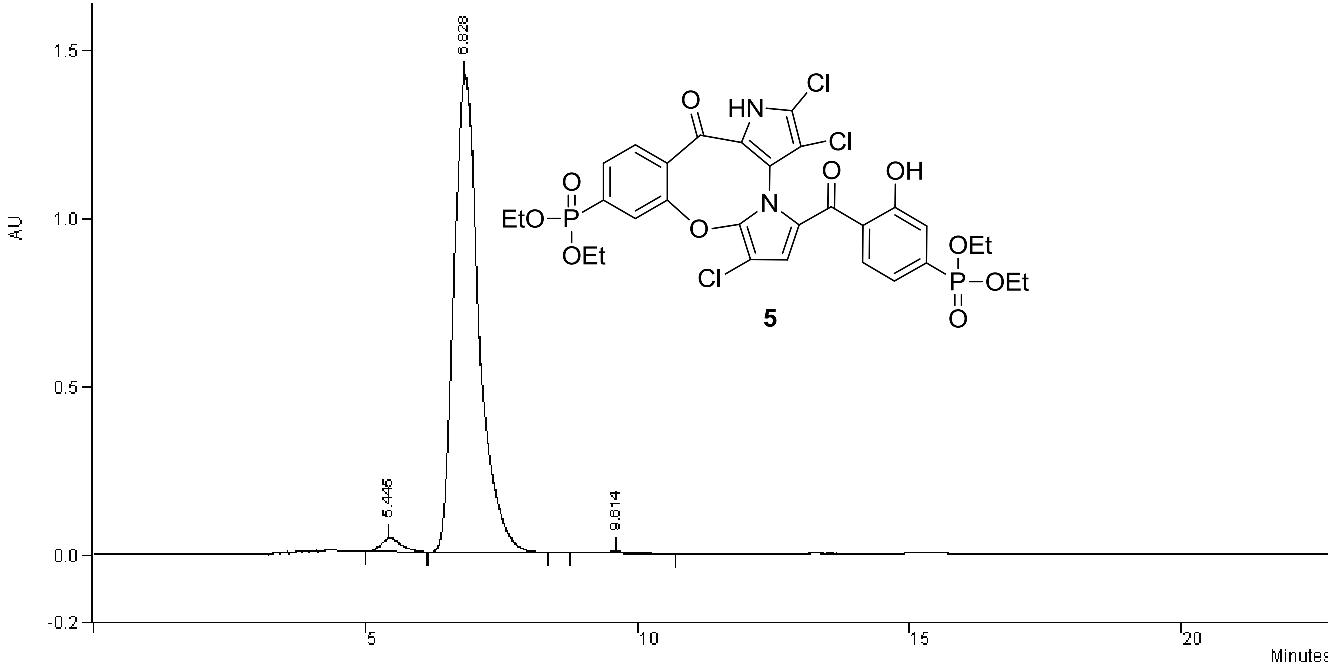


| Peak No. | Peak Name | Result () | Ret. Time (min) | Time Offset (min) | Area (counts) | Sep. Code | Width 1/2 (sec) |
|---|---|---|---|---|---|---|---|
| 1 | | 2.1044 | 5.445 | 0.000 | 9897132 | BB | 21.9 |
| 2 | | 97.2323 | 6.828 | 0.000 | 457290304 | BB | 28.2 |
| 3 | | 0.6633 | 9.614 | 0.000 | 3119744 | BB | 52.9 |
| | Totals: | 100.0000 | | 0.000 | 470307180 | | |

**Figure S28.** Purity of **5a** by HPLC.

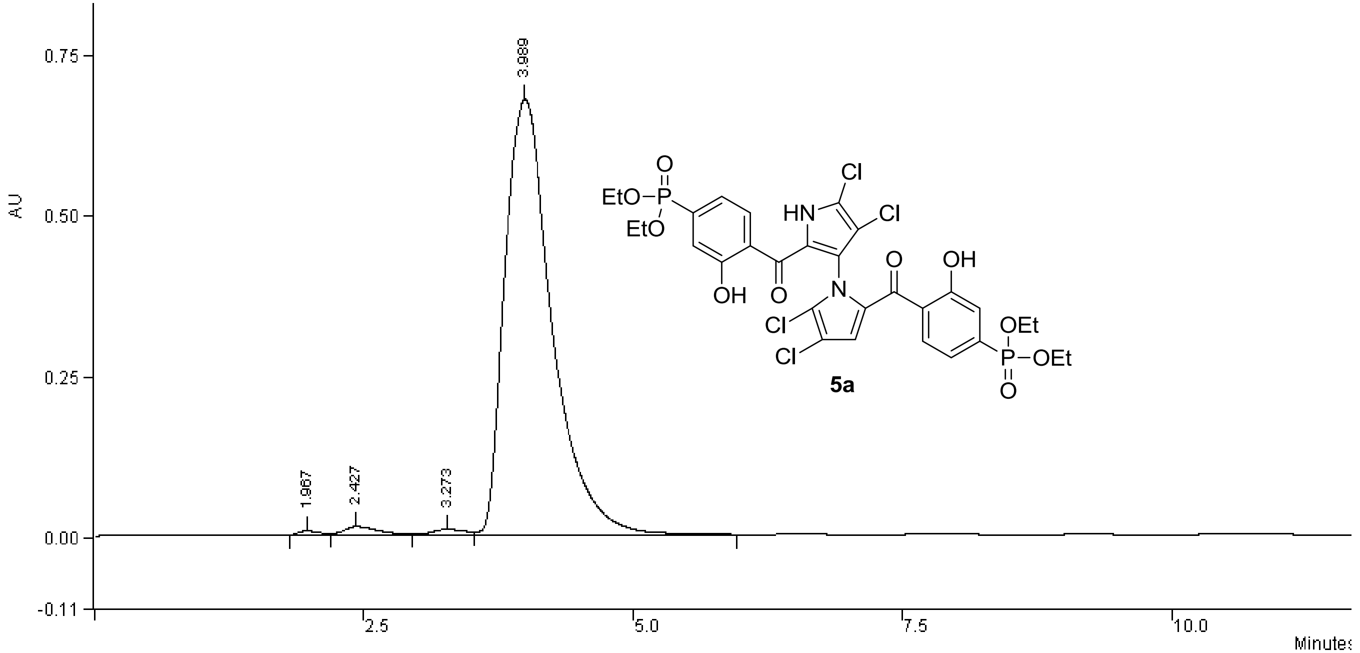


| Peak No. | Peak Name | Result () | Ret. Time (min) | Time Offset (min) | Area (counts) | Sep. Code | Width 1/2 (sec) |
|---|---|---|---|---|---|---|---|
| 1 | | 0.5003 | 1.967 | 0.000 | 1070013 | BV | 17.8 |
| 2 | | 1.5116 | 2.427 | 0.000 | 3232586 | VV | 21.7 |
| 3 | | 0.9578 | 3.273 | 0.000 | 2048307 | VV | 19.8 |
| 4 | | 97.0302 | 3.989 | 0.000 | 207502160 | VB | 27.8 |
| | Totals: | 99.9999 | | 0.000 | 213853066 | | |

**Figure S29.** Purity of **8** by HPLC.

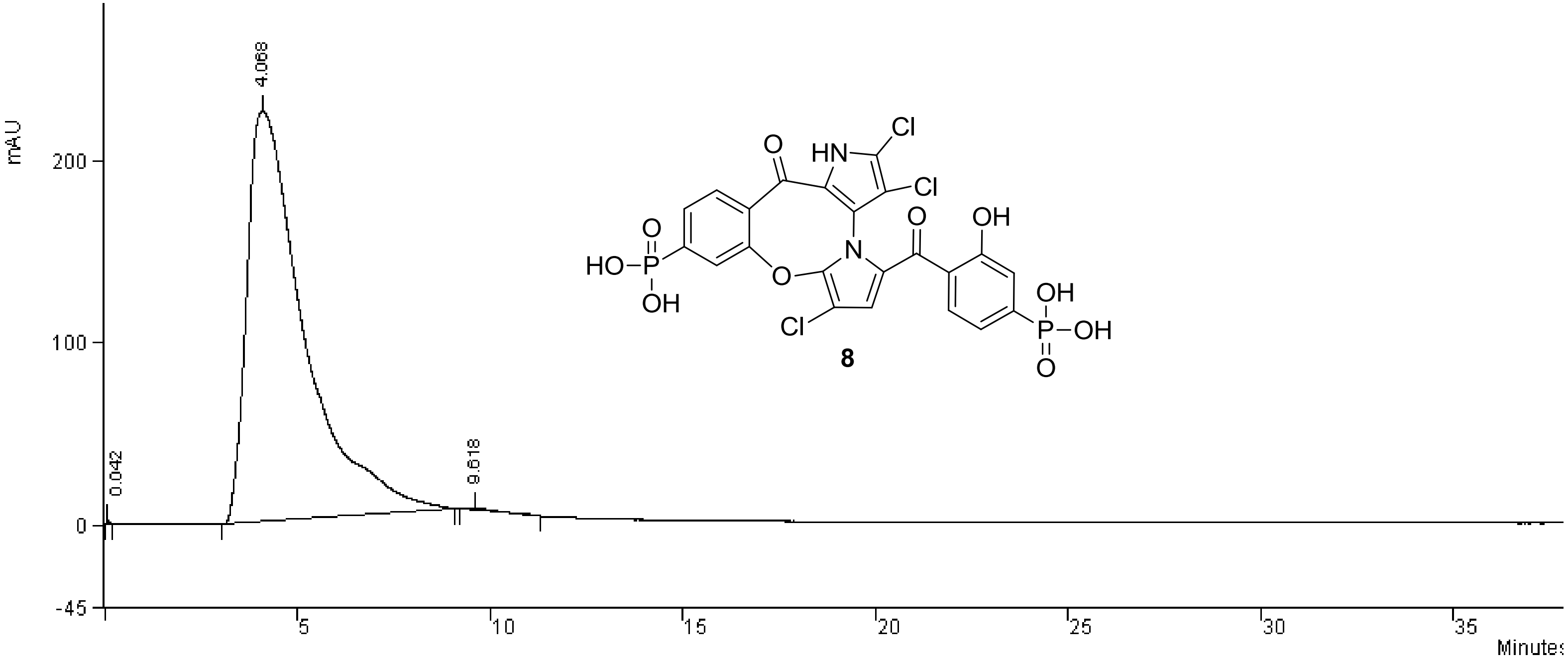


| Peak No. | Peak Name | Result () | Ret. Time (min) | Time Offset (min) | Area (counts) | Sep. Code | Width 1/2 (sec) |
|---|---|---|---|---|---|---|---|
| 1 | | 0.0206 | 0.042 | 0.000 | 48423 | BB | 3.4 |
| 2 | | 99.7386 | 4.068 | 0.000 | 234118784 | BB | 86.0 |
| 3 | | 0.2408 | 9.618 | 0.000 | 565247 | BB | 20.1 |
| | Totals: | 100.0000 | | 0.000 | 234732454 | | |

**Figure S30.** Purity of **8a** by HPLC.

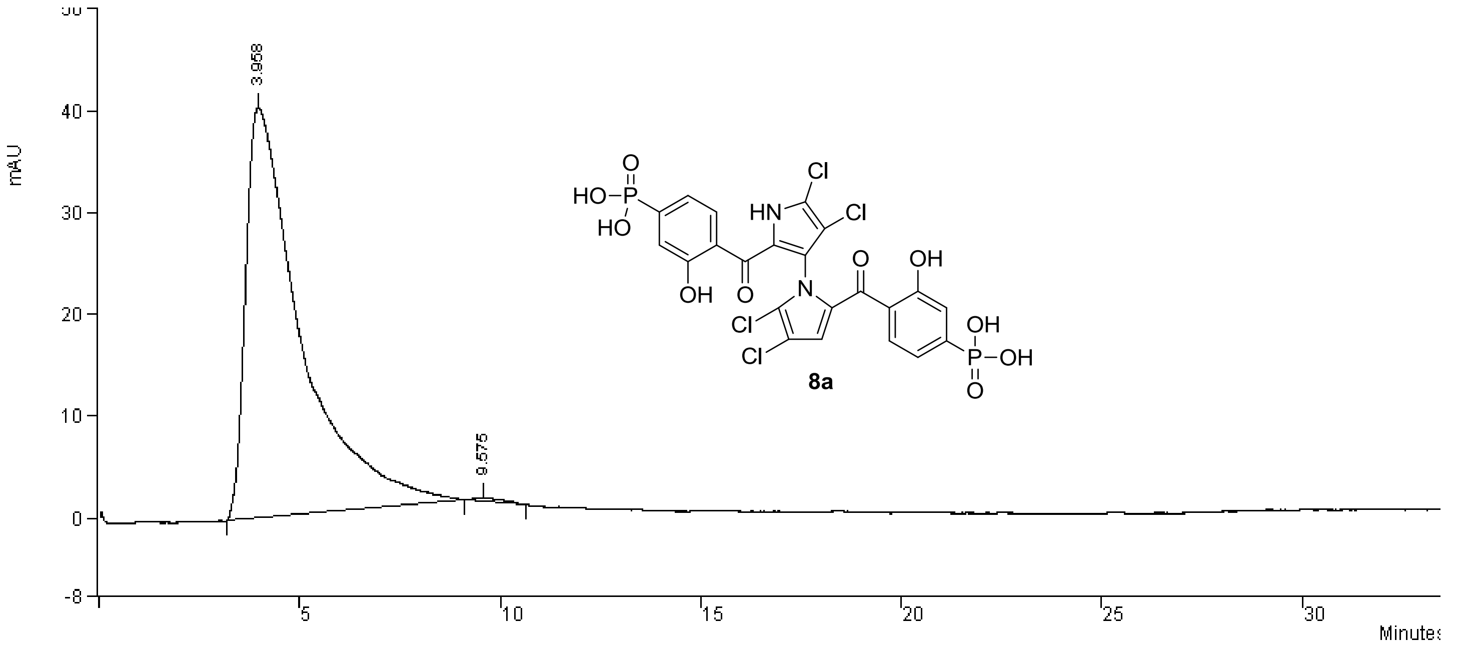


| Peak No. | Peak Name | Result () | Ret. Time (min) | Time Offset (min) | Area (counts) | Sep. Code | Width 1/2 (sec) |
|---|---|---|---|---|---|---|---|
| 1 | | 99.5090 | 3.958 | 0.000 | 38959564 | BB | 77.0 |
| 2 | | 0.4910 | 9.575 | 0.000 | 192223 | BB | 24.3 |
| | Totals: | 100.0000 | | 0.000 | 39151787 | | |

© 2014 by the authors; licensee MDPI, Basel, Switzerland. This article is an open access article distributed under the terms and conditions of the Creative Commons Attribution license (http://creativecommons.org/licenses/by/3.0/).
